# Plasma *N*-glycans in colorectal cancer risk

**DOI:** 10.1038/s41598-018-26805-7

**Published:** 2018-06-05

**Authors:** Margaret Doherty, Evropi Theodoratou, Ian Walsh, Barbara Adamczyk, Henning Stöckmann, Felix Agakov, Maria Timofeeva, Irena Trbojević-Akmačić, Frano Vučković, Fergal Duffy, Ciara A. McManus, Susan M. Farrington, Malcolm G. Dunlop, Markus Perola, Gordan Lauc, Harry Campbell, Pauline M. Rudd

**Affiliations:** 1 National Institute for Bioprocessing Research & Training, Dublin, Ireland; 2 Institute of Technology Sligo, Department of Life Sciences, Sligo, Ireland; 3 Centre for Global Health Research, Usher Institute for Population Health Sciences and Informatics, University of Edinburgh, Edinburgh, UK; 4 Colon Cancer Genetics Group, Institute of Genetics and Molecular Medicine, University of Edinburgh and Medical Research Council Human Genetics Unit, Edinburgh, UK; 5 Department of Medical Biochemistry and Cell Biology, Institute of Biomedicine, Sahlgrenska Academy, University of Gothenburg, Gothenburg, Sweden; 6 Pharmatics Limited, Edinburgh Bioquarter, 9 Little France Road, Edinburgh, UK; 7 Genos Glycoscience Research Laboratory, Zagreb, Croatia; 8 Department of Health, The National Institute for Health and Welfare, Helsinki, Finland; 9 University of Zagreb Faculty of Pharmacy and Biochemistry, Zagreb, Croatia; 10 Bioprocessing Technology Institute, Agency for Science, Technology and Research (A*STAR), 20 Biopolis Way, #06-01 Centros, Singapore, 138668 Singapore

## Abstract

Aberrant glycosylation has been associated with a number of diseases including cancer. Our aim was to elucidate changes in whole plasma *N*-glycosylation between colorectal cancer (CRC) cases and controls in one of the largest cohorts of its kind. A set of 633 CRC patients and 478 age and gender matched controls was analysed. Additionally, patients were stratified into four CRC stages. Moreover, *N*-glycan analysis was carried out in plasma of 40 patients collected prior to the initial diagnosis of CRC. Statistically significant differences were observed in the plasma *N*-glycome at all stages of CRC, this included a highly significant decrease in relation to the core fucosylated bi-antennary glycans F(6)A2G2 and F(6)A2G2S(6)1 (*P* < 0.0009). Stage 1 showed a unique biomarker signature compared to stages 2, 3 and 4. There were indications that at risk groups could be identified from the glycome (retrospective AUC = 0.77 and prospective AUC = 0.65). *N-*glycome biomarkers related to the pathogenic progress of the disease would be a considerable asset in a clinical setting and it could enable novel therapeutics to be developed to target the disease in patients at risk of progression.

## Introduction

Glycosylation is a highly prevalent and structurally diverse post translational modification which not only dictates the biological activity of proteins but influences cellular proliferation, inflammatory processes and metastasis^1,2^. Glycosylation is designated *N*-linked or *O*-linked depending if the glycosidic moiety occurs at Asn residues or at Ser/Thr residues, respectively^3–5^. Alterations due to physiological and pathophysiological conditions alter the ‘normal’ glycosylation profile. For example, the expression and activity levels of glycosidases and glycosyltransferases have a large influence on the abundance and distribution of glycans^6,7^. In a given physiological state, glycoform populations are reproducible; therefore, disease-associated alterations may provide diagnostic biomarkers. Analysis of the glycosylation pattern in biofluids such as serum or plasma may therefore be expected to provide useful biomarkers as the host responds to disease or as tumours secrete certain proteins^8,9^,. Previous research on lung^10,11^, breast^12–14^, ovarian^15,16^, periampullary^17^ and stomach^18^ cancer confirm the significance of the abundance of specific serum or plasma *N*-glycan structures (e.g. increased levels of sialyl Lewis X (SLe^X^)) to be hallmarks of cancer progression.

Colorectal cancer (CRC) is the 4th most commonly diagnosed cancer in UK (13% of all cancers) and the 2nd most common cause of cancer death (10% of total) (Cancer Research UK). In previous research we have found that CRC risk is linked to specific glycosylation changes in Immunoglobulin G (IgG) glycosylation, including a decrease in IgG galactosylation and IgG sialylation and an increase in core-fucosylation of neutral glycans with concurrent decrease of core fucosylation of sialylated glycans^19^. Subsequently, we have also reported that decreased galactosylation, decreased sialylation (of fucosylated IgG glycan structures) and increased bisecting GlcNAc in IgG glycan structures were strongly associated with all-cause and CRC mortality^20^.

In this study a high-throughput (HTP) automated ultra-performance liquid chromatography (UPLC)-fluorescent method was used to examine the plasma *N*-glycome from over one thousand patients diagnosed with CRC and significant cancer associated *N*-glycan alterations were identified in relation to CRC risk. Glycoproteins account for the majority of plasma proteins and the contribution of the highly abundant glycoproteins to the prevailing serum/plasma *N*-glycome have previously been documented^21,22^. It has been shown that many of these glycoproteins are implicated in diseases such as cancers, autoimmune disease and congenital disorders of glycosylation. Therefore by measuring the relative abundance of glycans released from these glycoproteins potential diagnostic biomarkers could be revealed and targeted treatments employed^21^. UPLC hydrophilic interaction liquid chromatography with fluorescence detection (HILIC)^23,24^ was utilized to characterise the glycosylation profile of plasma samples from 633 CRC patients and 478 matched healthy population controls. The differences in relative abundance of glycans between cases and controls and the determination of correlations in the context of body mass index (BMI), C-reactive protein (CRP), family history, non-steroidal anti-inflammatory drug (NSAID) intake, physical activity and smoking habits were investigated. Importantly, we also included CRC stage results in our analysis. Finally, the predictive value of the relative glycan abundances to discriminate CRC from healthy control and identify at risk individuals using optimized classification algorithms was assessed.

## Results

**Figure 1:**
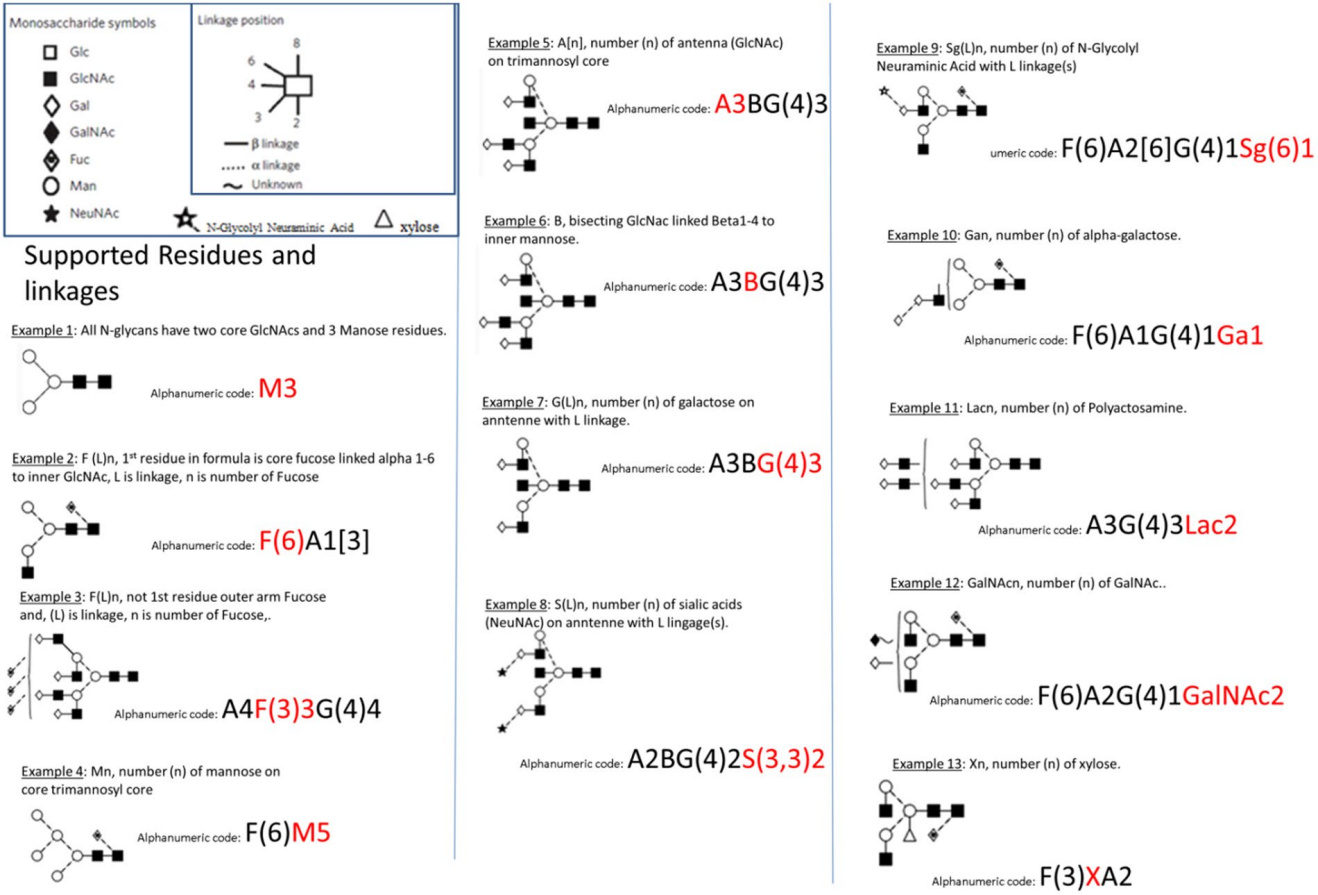
The Oxford notation by example. Inset: the most common residues in *N*-glycans and their linkages. Note that linkage information may be left ambiguous e.g. F(6)A1[3] may be written FA1.

**Figure 2:**
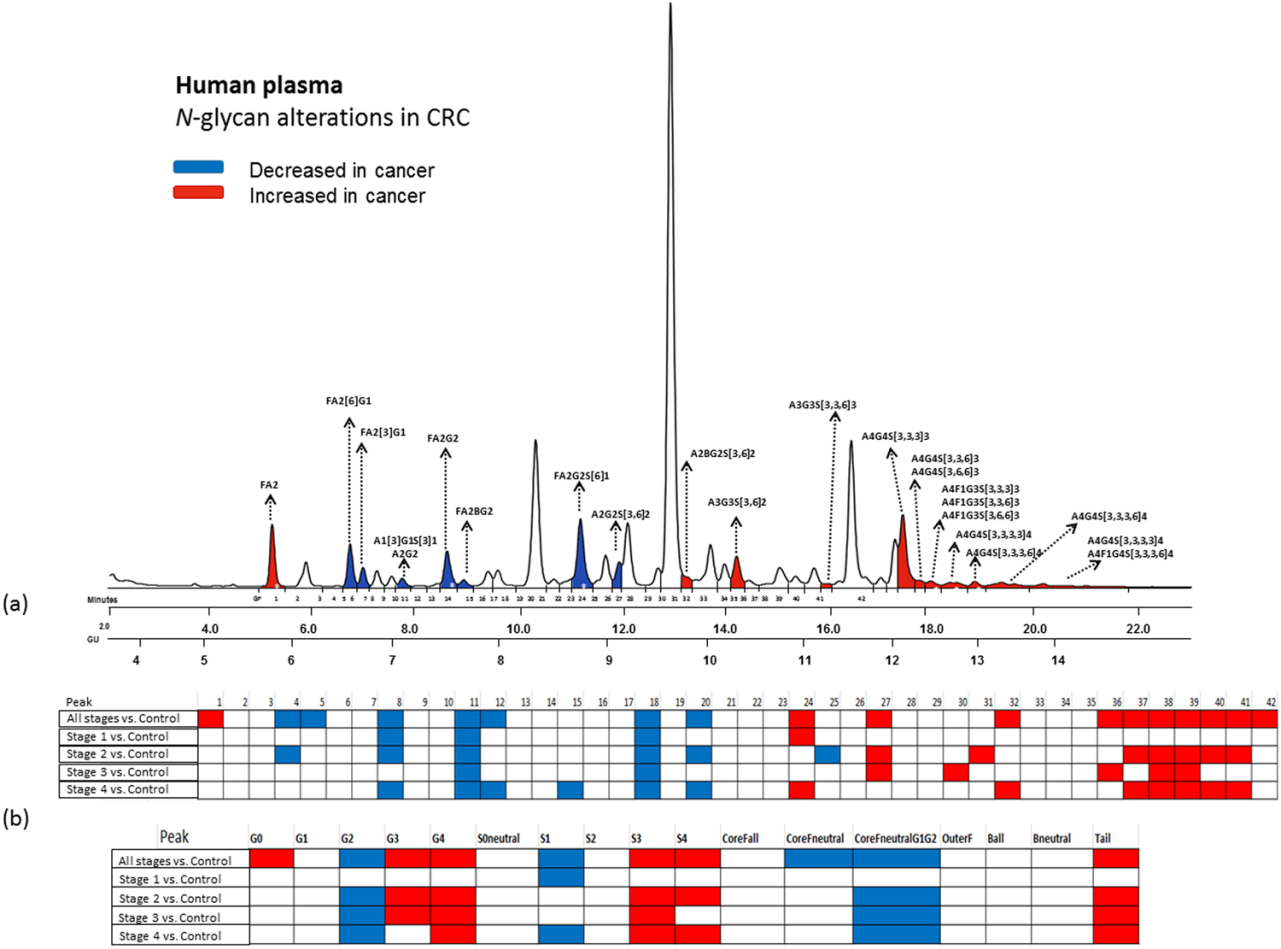
(**a**) A representative chromatogram from human plasma *N*-glycome and peak assignments from the CRC cohort. Significant peaks (found on training set of 625 patients vs. 468 control) are coloured in red (increased in CRC) and blue (decreased in CRC). ‘*’ indicates one of the top five peak abundance changes (i.e. lowest p-value). (**b**) Significant peaks are marked decreased (blue) or increased (red) in all CRC and four stages of CRC.

Total plasma *N-*glycan composition was determined by UPLC analysis of 2AB-labelled glycans as reported for HPLC^25^ and more recently for UPLC where Saldova *et al*.^12^ carried out extensive glycan analysis using exoglycosidase sequencing and MS to confirm all structures and populate a database. The chromatogram was integrated into 42 distinct individual peaks. The Oxford nomenclature has been used to annotate individual glycan structures^26^ where A represents the number of antennae present, F indicates the fucose, B indicates the presence of a bisecting *N*-acetylglucosamine, G represents galactoses and S denotes sialic acids (Fig. 1). A representative chromatogram showing separation of the plasma glycome into predominant structures is shown in Fig. 2a and Supplementary Fig. 1. Each glycan identified, its peak number and GU value is shown in Supplementary Table 1.

### Clinical features

The distribution and significant clinical features for the CRC patients and healthy controls is summarized in Table 1. Family history of CRC cancer was statistically significantly different between CRC cases and controls (*P*-value = 3.81E-39). Other categorical features such as smoking status, physical activity, gender and NSAIDs intake showed no statistical significance in this subset of the SOCCS study. However, NSAIDs intake was approaching significance (*P* = 6.62E-02) but did not pass Bonferroni correction. Plasma levels of CRP were found to be statistically significantly increased (*P* = 8.61E-11) in CRC. Interestingly, low BMI was associated with a low risk of CRC (*P* = 1.31E-0.7). Only the statistically significant variables of CRP, BMI and cancer family history were analysed further to explore their associations with the glycan traits.

**Table 1:** Descriptive information for 625 CRC patients vs. 468 healthy controls.

Feature	CRC (n = 625)	Control (n = 468)	P-value
Age (median[IQR])	52 (47–55)	53 (48–56)	2.32E-02
BMI (median[IQR])	26 (23–29)	28 (26–31)	*1.31E-0.7*
CRP (median[IQR])	0.36 (0.12–1.635)	0.18 (0.09–0.60)	*8.61E-11*
KCalories (median[IQR])	2527 (2006–3366)	2572 (2055–3238)	4.63E-01
Gender (male/female)	54.24/45.76	55.34/44.66	7.59E-01
Family history (Low/medium or high/unknown)	67.84%/27.04%/5.12%	96.03%/1.05%/2.93%	*3.81E-39*
Physical activity (very low or low/medium or high/unknown)	58.08%/14.08%/27.84%	62.18%/14.96%/22.86%	3.49E-01
Smoking status (non/current or former/unknown)	36.96%/37.44%/25.60%	38.25%/41.03%/20.73%	2.62E-01
NSAIDs (no/yes/unknown)	60.00%/15.04%/24.96%	59.62%/19.66%/20.73%	6.62E-02

Underlined p-values show significant differences between CRC and control. To highlight biological variation for continuous variables the median and interquartile ranges (IQR) are shown. IQR shows the spread of the continuous variables. For categorical features the basic counts are shown.

### Significant peaks, glycans and glycan groups

Statistically significant differences were observed for 18 out of the 42 glycan peaks in the chromatogram. Supplementary Table 2 shows the differential analysis of each peak with their *P*-values. Figure 2a summarizes the statistically significantly increased and decreased predominant glycans, their retention times (RTs), GUs and relative abundance (i.e. peak areas) on the chromatogram. Except for the first peak, the significant glycans eluting at earlier retention times (pre major peak 23) were consistently decreased in CRC, while the later eluting glycans were consistently increased (post major peak 23). Of the peaks decreased in CRC, the majority had core fucosylated structures and all were bi-antennary, contained mono or di-galactosylated moieties and at most one involving sialic acid. The most statistically significant decrease came from the F(6)A2G2 (GP11) glycan (*P* = 2.00E-16). Increased in CRC were glycans containing 2 or more sialic acid and galactose residues. In the tail end region of the chromatogram the majority CRC abundant glycans came from highly branched structures (>3 GlcNAc antennae), highly galactosylated (>3 galactose) and highly sialylated (>3 sialic acids) glycans. The most statistically significant increase (*P* = 2.00E-16) came from the tetra-antennary structure A4G4S[3,3,3,3]4 (GP39). All isomers of A4G4S4 were consistently increased in CRC.

### Cancer stage and biomarkers

Figure 2b shows the significant biomarker signatures across the four stages of CRC. A decrease of peak 11 (F(6)A2G(4)2) and peak 18 (F(6)A2G(4)2 S(6)1) is a constant biomarker across all stages of CRC. However, stage 1 has an evidently different biomarker signature compared to stages 2, 3 and 4. That is, stage 1 has no significant increase in the tail region (peaks 36–42).

### Grouping glycans by structural features

**Figure 3:**
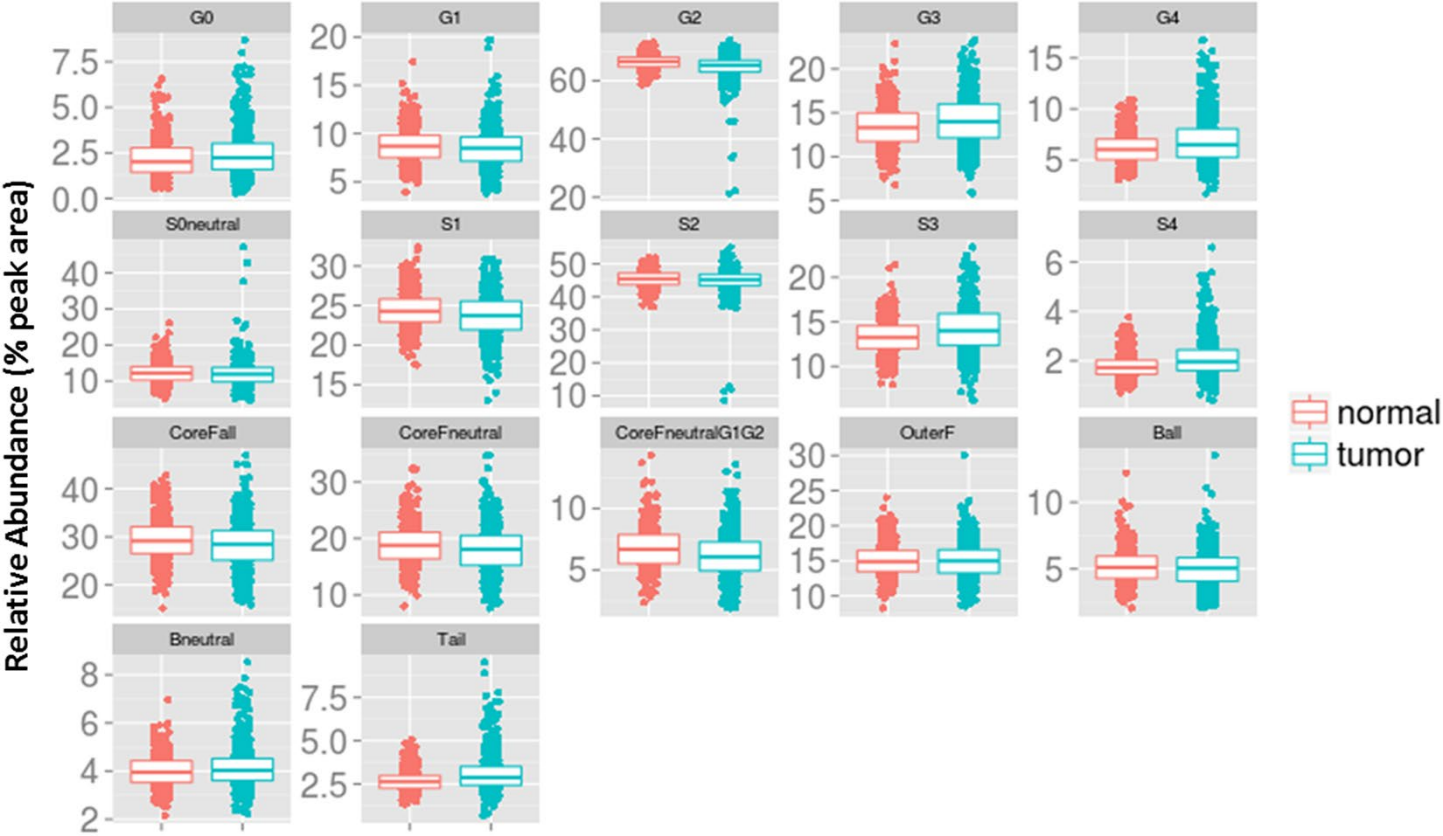
Boxplots showing increased and decreased glycan abundance for the derived glycome traits. The dots represent an individual’s relative abundance for the trait. Statistically significant traits can be found in Table 2.

Many of the individual structures share the same structural features: (galactose (G), sialic acid (S) and core-fucose (F)), thus additional derived traits were calculated that average these features across multiple glycans (Supplementary Table 4). Significant differences were observed in several features of the derived glycome traits (Table 2, Fig. 3). The first peak, GP01, containing the only non-galactosylated glycan (G0) was increased in CRC (P = 1.41E-04 for G0; Table 2; Fig. 3). When there was galactosylation, only di-galactosylation was significantly decreased (*P* = 1.27E-11 for G2; Table 2; Fig. 3), while a concurrent increase in tri and tetra galactosylation was observed (*P* = 6.25E-10 for G3 and *P* = 2.87E-08 for G4; Table 2, Fig. 3). Neutral and low branching sialylation (S0 neutral, S1 and S2) were continuously decreased in CRC, but only S1 was significantly so (*P* = 4.94E-06 S1; Table 2 and Fig. 3). There was a significant switch to an increased abundance in CRC for tri and tetra sialylation (*P* = 2.37E-11 for S3; and *P* = 2.74E-06 for S4; Table 2 and Fig. 3) with end RTs. In particular, the last seven eluting peaks containing tri/tetra-sialylated glycans collectively showed significance (*P* = 9.94E-09 for Tail in Table 2; Fig. 3). Core fucosylation was decreased in CRC when the glycans were neutral, and more so when neutral with one or two galactose: i.e. *P* = 1.51E-03 for coreFneutral Table 2 and *P* = 3.57E-09 for coreFneutralG1G2 Table 2. Finally, grouping bisecting or outer fucosylated peaks showed insignificant change in abundance between CRC and control (Table 2; Fig. 3). The relationship between the specific glycan biomarkers and the derived biomarker traits are summarized in supplementary Table 5. All predominant potential *N*-glycan biomarkers in CRC plasma with potential glycoproteins involved are displayed in Table 3 and a comparison to the previous IgG analysis is outlined.

**Table 2:** Plasma glycome composition in CRC patients and controls. Only the main derived traits describing glycome composition are shown.

Glycan trait	CRC (n = 625) (median[IQR])	Control (n = 468) (median[IQR])	Δ peak area*	p-values
G0	2.23 (1.59–3.02)	2.01 (1.44–2.78)	0.29	1.41E-04
G1	8.49 (7.14–9.63)	8.695 (7.5–9.8175)	−0.22	1.99E-02
G2	65.34 (63.04–66.95)	66.6 (64.82–68.05)	−1.79	1.27E-11
G3	13.97 (12.14–15.99)	13.295 (11.7225–14.98)	0.63	6.25E-10
G4	6.5 (5.26–8.05)	6.02 (5.03–7.0375)	0.81	2.87E-08
S0neutral	11.91 (9.79–13.73)	12.215 (10.2075–14)	−0.18	1.13E-01
S1	23.72 (21.95–25.56)	24.29 (22.9125–25.825)	−0.72	4.94E-06
S2	45.15 (43.41–46.83)	45.465 (43.785–47.14)	−0.49	7.89E-01
S3	13.99 (12.36–15.92)	13.24 (12.005–14.5475)	0.93	2.37E-11
S4	1.96 (1.6–2.44)	1.72 (1.45–2.02)	0.34	2.74E-06
CoreFall	28.43 (25.16–31.28)	29.135 (26.535–32.105)	−1.06	4.30E-03
CoreFneutral	18.07 (15.23–20.52)	18.78 (16.4025–21.095)	−0.89	1.51E-03
CoreFneutralG1G2	6.05 (4.93–7.28)	6.685 (5.515–7.8675)	−0.65	3.57E-09
OuterF	14.98 (13.27–16.58)	14.885 (13.47–16.46)	0.07	6.35E-01
Ball	5.06 (4.08–5.85)	5.115 (4.29–5.9575)	−0.16	2.58E-01
Bneutral	4.03 (3.61–4.52)	3.95 (3.53–4.43)	0.15	3.46E-02
Tail	2.88 (2.43–3.51)	2.63 (2.26–2.98)	0.46	9.94E-09

Directly measured glycan structures are available in Supplementary Table 1. Description of each derived trait is given in Supplementary Table 4. *Bonferroni* correction for multiple testing (*P*-values significance threshold <0.05/17 (0.003). *The difference between the mean peak areas (CRC – control). ^#^Results reported on this column. G – galactose; F – fucose; B – bisecting GlcNAc, S – sialic acid. To highlight biological variation the median and interquartile ranges (IQR) are shown. IQR shows the spread of the relative abundances (i.e. summed peak areas).

**Table 3:** Comparison between the IgG glycan markers^19^ to the plasma glycan markers found in this work.

Predominant Glycan	Plasma CRC change	This work’s Peak	IgG change CRC^19^	IgG Peak from^19^	Likely plasma protein(s)*	Amino acid	Protein plasma concentration (% of total)^
FA2	Increased	GP01	Increased	GP4	IgG	Asn297, Asn322 (IgG3)	40.4%
FA2[6]G1	Decreased	GP04	Decreased	GP08	IgG	Asn297, Asn322 (IgG3)	40.4%
FA2[3]G1	Decreased	GP05	Decreased	GP09	IgG	Asn297, Asn322 (IgG3)	40.4%
A2[3]G1S[3]1	Decreased	GP08	NF	NF	?	?	?
A2G2	Decreased	GP08	Decreased	GP12	IgG, Apo	Asn297 (IgG), Asn322 (IgG3)	40.4% (IgG), 0.5% (Apo)
FA2G2	Decreased	GP11	Decreased	GP14	IgG	Asn297 (IgG), Asn322 (IgG3)	40.4%
FA2BG2	Decreased	GP12	Decreased	GP15	IgG	Asn297 (IgG), Asn322 (IgG3)	40.4%
FA2G2S1	Decreased	GP18	Decreased	GP18	IgG, IgA, IgE, IgD, IgM, A2M	Asn297 (IgG), Asn322 (IgG3), Asn340(IgA), Asn46(IgM), Asn209(IgM), Asn272(IgM)	40.4% (IgG), 9.0% (IgA), 0.1% (IgD), 5.0% (IgM)
A2F1G2S1	Decreased	GP20	NF	NF	Apo D, Haptoglobin	Asn98 (Apo D), Asn184(Hapto), Asn207 (Hapto), Asn241 (Hapto)	0.3% (ApoD), 4.5% (Hapto)
A2BG2S2	Increased	GP24	Decreased	GP22	IgG	Asn297 (IgG), Asn322 (IgG3)	40.4%
FA3G3S1	Increased	GP24	NF	NF	?	?	?
FA3BG3S1	Increased	GP24	NF	NF	?	?	?
A3G3S2	Increased	GP27	NF	NF	B2, Apo D, Hapto, Sero	Asn65 (Apo D), Asn162 (B2), Asn193 (B2), Asn184 (Hapto), Asn211 (Hapto), Asn241 (Hapto), Asn432 (Sero), Asn630 (Sero)	0.7% (B2), 0.3% (Apo D), 4.5% (Hapto), 8.5% (Sero)
A3BG3S2	Increased	GP27	NF	NF	?	?	?
FA3G3S3	Increased	GP32	NF	NF	?	?	?
A4G4S3	Increased	GP37	NF	NF	AGP	Asn72, Asn93, Asn103	2.6% (AGP)
A4F1G3S3	Increased	GP38	NF	NF	AGP	Asn93	2.6% (AGP)
A4G4S4	Increased	GP42	NF	NF	AGP, Apo D, CP	Asn65 (Apo D), Asn762 (CP)	2.6% (AGP), 1.2% (CP), 0.3% (ApoD)
A4F1G4S4	Increased	GP42	NF	NF	AGP, CP	Asn762 (CP)	2.6% (AGP), 1.2% (CP)

*The possible plasma protein and amino acid site involved for each glycan derived from^21^ and the previous IgG CRC study^19^, in bold the highest concentration. NF: not found in the IgG profile. For simplicity we did not separate by linkage isomers since all followed the same trend (e.g. A4G4S[3,3,3,3]4 and A4G4S[3,3,3,6]4 both increased in plasma CRC). Abbreviated proteins (UniProt ID)- AGP: Alpha-1-acid glycoprotein (P02763; P19652), A2M: Alpha-2-macroglobulin (P01023), Apo: Apolipoprotein B-100 (P04114), Apo D: Apolipoprotein D (P05090), CP: Ceruloplasmin (P00450), Hapto: Haptoglobin (P00738), B2: Beta-2-glycoprotein I (P02749). Sero: Serotransferrin (P02787). IgA: Immunoglobulin A (P01876, P01877), IgD: Immunoglobulin D (P01880), IgE: Immunoglobulin E (P01854), IgG: Immunoglobulin G (P01857, P01859, P01860, P01861), IgM: Immunoglobulin M (P01876, P01877), ?: unknown. ^Approx. derived from^21^.

### Glycan abundance changes related to CRP, BMI and family history

Higher abundance of the peak cluster (GP36, GP37, GP38, GP39, GP40, GP41 and GP42) containing mainly tri/tetra galactose/sialic acid glycans was found to be highly correlated with CRP levels (see Pearson correlations in Supplementary Table 6). These peaks, which were found to be increased in CRC (see Fig. 2a, Table 2 and Supplementary Table 2) could therefore be considered markers for inflammation too. Conversely, core fucosylated peaks GP11 and GP18 were inversely correlated with CRP (Pearson 95% CIs: [−0.33, −0.25] and [−0.32, −0.24] Supplementary Table 6) thus higher abundance of FA2G2 and FA2G2S[6]1 glycans were indicators of normal body or little inflammation. BMI did not show any interesting correlations with glycan peak abundances. Interestingly, individuals with high or medium family history had significantly increased GP40 (*P* = 1.65E-03; Supplementary Table 6 and Supplementary Fig. 2).

### Discriminating CRC cases from healthy controls

#### Discrimination models

**Figure 4:**
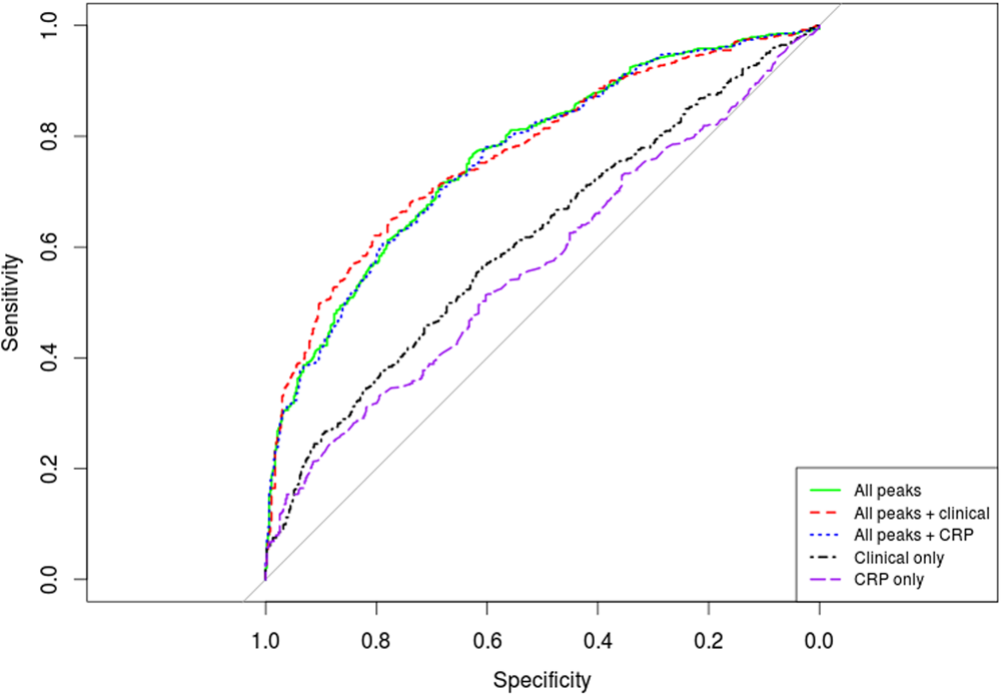
Classification of CRC patients using plasma glycans. ROC curve illustrating the performance of perceptron model in discrimination between CRC patients and healthy controls from SOCCS retrospective study (625 CRC vs. 468 control). “All peaks’: 42 peak areas. ‘All peaks + clinical’: 42 peak areas, BMI, age, gender, smoking status, physical activity, NSAIDs intake, Kcalorie intake and CRP. All peaks + CRP: 42 peak areas and CRP only. ‘Clinical only’: BMI, age, gender, smoking status, physical activity, NSAIDs intake, Kcalorie intake and CRP. ‘CRP only: the only variable used is CRP’.

We also optimized a simple machine learning algorithm to model the observed separation in the training data (625 CRC vs. 468 control). The optimized discrimination models were evaluated in a ten-fold cross validation. Clinical variables such as age, gender, smoking status, BMI, CRP, physical activity and Kcalories were available in substantial numbers across both CRC cases and controls and therefore could be used in the optimization (see Table 1). However, cancer family history could not be used, as there were only 5 cases of high/medium family history in controls and they would not be representative for model optimization. Table 4 shows that using all glycan peak areas (without derived traits) as input, an Area Under the ROC Curve (AUC) of 0.765 was possible. Model performance of glycan and clinical features resulted in an improvement in AUC by 1% point (0.777 vs. 0.765; *P* = 9.48E-02; Table 4). We thought that CRP may be an interesting clinical feature to combine alone with all peaks, however using all peaks + CRP showed very little improvement over simply using all peaks alone (Table 4; AUC 0.763 vs. 0.765). Clinical features (age, gender, smoking status, BMI, CRP, physical activity and Kcalories) alone had only modest performance (AUC 0.613; *P* < 0.0001) suggesting the glycan information quantified in the peak areas is more discriminative at predicting colorectal cancer than any of the clinical features. CRP alone had almost random classification AUC (Table 4; AUC 0.569) suggesting it needed to be used in combination with other clinical features (Table 4; CRP AUC vs. Clinical only AUC; 0.569 vs. 0.613). Figure 4 shows the ROC curves for the best models in Table 4 compared to clinical features and CRP only baselines. By design our models were tuned to produce a specificity of approximately 0.95 (5% false positive rate). This is because for cancer screening, where the target cancer has low occurrence in the screened population, the false positive rate needs to be very low^27^. At this 0.95 specificity the two models using all peaks alone and clinical features alone achieved a sensitivity of 0.3444 and 0.1343 respectively (Table 4). This improved by 2% points to 0.3649 sensitivity (at ~0.95 specificity) when combining clinical and all glycan peak features (Table 4).

**Table 4:** 10-fold cross validation model performance to identify CRC.

Peaks + features	AUC	Sensitivity	Specificity	p-value
All peaks	0.765	0.318	0.949	9.8E-02
All peaks + clinical	0.770	0.373	0.949	*
All peaks + CRP	0.763	0.322	0.949	8.93E-02
Clinical only	0.613	0.150	0.949	<0.0001
Clinical only - CRP	0.529	0.075	0.949	<0.0001
CRP only	0.569	0.153	0.949	<0.0001

Discrimination models on the SOCCS dataset (625 CRC vs. 468 control). ‘All peaks’: 42 peak areas. ‘All peaks + clinical’: 42 peak areas, BMI, age, gender, smoking status, physical activity, NSAIDs intake, Kcalorie intake and CRP. ‘All peaks + CRP’: 42 peaks areas + CRP only. ‘Clinical only’: BMI, age, gender, smoking status, physical activity, NSAIDs intake, Kcalorie intake and CRP. ‘Clinical – CRP’ used all clinical features except family history and CRP. *P*-value column: ‘*’ best model, *P*-value AUC different from best model.

Furthermore, shown in Supplementary Table 7, all stages could be discriminated from healthy control with high AUC (stage 1: 0.777, stage 2: 0.781, stage 3: 0.781 and stage 4: 0.840). As one might expect stage 4 had the highest discrimination power (AUC 0.840, sensitivity 0.5287 and specificity 0.9477). More importantly, stage 1 had a high discrimination power (AUC 0.777, sensitivity 0.3482 and specificity 0.9477) suggesting the glycome can also detect early stage CRC.

### Validating the discrimination model

All parameters of the discrimination model were optimized on the training set (625 CRC cases vs. 468 control). Then, the model was used to predict the probability of CRC on 18 individuals in the validation set (8 CRC vs. 10 control). As we derived the cut-off threshold on the training set at 95% specificity we expected highly accurate healthy control detection. Supplementary Table 8 shows that using peak area information alone none of the 10 healthy controls were classified as CRC while 4 of the CRC patients were flagged as cancer. In total, this test produced an accuracy of 77.8%, with a 100% and 50% specificity and sensitivity respectively. Adding the clinical variables produced a sensitivity of 62.5% and a specificity of 90.0%. CRP alone degraded the sensitivity greatly suggesting again that richer information is present in the relative abundance of the N-glycans (i.e. peak areas).

*At risk groups:* As mentioned previously, high/medium family history contained higher levels of peak 40 (predominantly glycan A4G4S[3,3,3,3]4) and therefore may represent a biomarker to identify at risk groups. In addition, the same glycan peak (A4G4S[3,3,3,3]4) was increased in individuals who were sampled before they developed colorectal cancer (FINRISK dataset; *P* = 1.10E-03; Supplementary Table 3). Further evidence that at-risk groups can be identified was confirmed by using all peak areas and age (allowed as both at risk and control groups were age matched) of individuals in our classification algorithm showing some discrimination power to identify at risk individuals (FINRISK dataset; Table 5; AUC 0.651).

**Table 5:** 10-fold cross validation model performance to identify at risk groups.

Peaks + features	AUC	Sensitivity	Specificity	p-value
All peaks + age	0.651	0.125	0.950	*
All peaks	0.612	0.150	0.950	<0.0001

Discrimination models tested on the FINRISK dataset. ‘All peaks’: 39 peak areas and ‘All peaks + age’: all 39 peak areas and age of each person in the FINRISK dataset. *P*-value column: ‘*’ best model, *P*-value AUC different from best model.

## Discussion

In this extensive comprehensive study, glycoforms were analysed in plasma from colorectal cancer patients and we assessed the correlation between specific glycan structures with associated CRC risk factors. Current CRC screening and surveillance strategies include colonoscopy, barium enema, sigmoidoscopy and faecal occult blood testing^28^. Using patient serum or plasma represents a much less invasive alternative and may improve patient compliance. Glycans in plasma are pooled from all cells in the body, and specific oligosaccharides produced during disease pathogenesis will be present and have already been directly correlated to various cancers^11,29^, and inflammatory conditions^30,31^. Bones *et al*. previously evaluated the contribution of glycosylation present on four highly abundant glycoproteins to the glycosylation profile of serum. The *N*-glycans displayed on IgG, transferrin, haptoglobin and α1-acid glycoprotein corresponded to the majority of peaks present in the serum *N*-glycome^22^. A recent review detailed the glycoforms present on 24 glycoproteins which account for the majority (30mg/mL-70–75mg/mL approximately) of the total plasma protein concentration^21^. Consequently, changes in the total plasma glycome are conceivably due to alterations in the glycosylation present in one or more of these highly abundant glycoproteins. In this study glycans were removed from their respective glycoproteins in the plasma samples by enzymatic digestion prior to separation by HILIC chromatography. Each glycoform was then quantified in a relative manner. Thus, by comparing CRC patient samples against controls, we can identify glycan biomarkers and postulate from which of the 24 plasma glycoproteins they originated. By analysing and comparing 625 CRC patients and 468 matching controls, we have found that CRC is associated with five major alterations in plasma glycome composition: (i) agalactosylation - glycans with no galactose residues were increased in CRC (ii) galactosylation – there was a decrease in mono and di-galactosylated structures and an increase in tri and tetra-galactosylated glycans, (iii) sialylation - a decrease was observed in mono-sialyated glycans and an increase in tri and tetra-sialylated structures, (iv) GlcNAc antennae - a decrease in galactosylated and sialylated bi-antennary GlcNAc glycans and an increase in highly branched (≥3 GlcNAc antenna) glycans (v) core fucose - a decrease in neutral core fucosylated glycans in particular ones with one or two galactose residues. The glycans which increased in CRC (all –tri and tetra antennary, heavily sialylated and galactosylated structures) are mainly found on the plasma protein α−1-acid (AGP)^21^. AGP is an acute phase protein predominantly synthesized in hepatic parenchymal cells and is known to be augmented in inflammatory responses. The increase observed in this study correlates with a previous study which documented that AGP contributes bi-antennary di-sialylated, tri-antennary tri-sialylated and tetra-antennary tetra sialylated glycans to the serum *N*-glycome in stomach cancer^22^. A statistically significant positive correlation was found between AGP and SLe^x^ expression in CRC and it is thought that the glycoprotein may be a possible carrier of SLe^x^^32^. Moreover, a positive correlation was demonstrated between increased levels of these highly branched, sialylated and galactosylated glycans with circulating levels of CRP (discussed below). Conversely, the decreased glycans (core fucosylated, mono or bi galactosylation and mono-sialylation) appear to be derived from IgG and to a lesser extent IgM^21^.

Glycan Peak 1 was atypical since it was the only glycan with a low mass/retention time which displayed an increase in abundance in CRC. In particular, this peak contained predominantly a core-fucosylated agalactosylated glycan (FA2). This concurs with previous studies which found increased levels of FA2 in ovarian cancer^16,29^, early stage breast cancer^33^ and stomach cancer^18^ with increasing disease pathogenesis. This was the only peak with a non-galactosylated component in our sample set and can be associated with IgG in plasma^21^. Furthermore, its abundance was also found to be increased in CRC in our previous IgG study^19^.

Specific glycans which were decreased in CRC were those containing either one sialic acid, one or two galactose and/or a core fucose included: FA2[6]G1, FA2[3]G1, A2[3]G1S[3]1, A2G2, FA2G2, FA2BG2, FA2G2S[6]1. An increase in expression of FUT8 (responsible for the transfer of a core fucose residue to *N*-linked oligosaccharides) in CRC has been described previously^19^. Additional support for FUT8 involvement is found in reports that mRNA MiR-198 represses tumour growth and metastasis in CRC by targeting FUT8^34^. Highly branched glycans containing tri and tetra sialyated and galactosylated residues were found to increase in CRC and included isomers of: A4G4S4, A4F1G3S3 and A4G4S3 likely originating from the AGP glycoprotein^21^. Importantly, previous comprehensive analysis utilizing exoglycosidase sequencing^12^ enabled definition of isomeric biomarkers particularly important for specifying sialic acid linkages (e.g. A4G4S[3,3,6]3 as opposed to the more general A4G4S3 form).

A large group of consecutive peaks (36–42) with increased glycan abundance in CRC cases were termed the “Tail” peaks as they all occurred at the later retention times of the chromatogram. These glycan peaks consisted of tri and tetra sialyated and galactosylated glycans lacking a core fucose. GP38 and GP42 contained outer fucose. This increase in tri and tetra antennary structures containing tri-sialylated and tetra-sialylated glycans agree with previous findings observed in other cancers such as lung^10^, breast^12^, periampullary^17^, ovarian^16^, stomach^18^ and breast with higher circulating tumour cell count^13^. It was hypothesized that the reason for the increase was this region contained, in part, the SLe^x^ epitope^10,13,16^. In addition, our results and that of previous studies^17^ confirmed CRP or inflammation levels were positively correlated to this particular region of the glycosylation profile. These SLe^x^ containing glycoforms were shown to be elevated in acute phase proteins^16^. We can therefore hypothesize that the glycan abundances here mainly originate from the AGP acute phase glycoprotein^21^. Additional evidence for tri and tetra sialylated glycoforms points towards altered expression of the enzyme ST6Gal-I in various cancers thus increasing levels of tri and tetra α2–6 sialic acid glycans^35,36^.

Categorising glycans by specific properties allowed a concise description of the global features which are up and down regulated in CRC. When analysing the peaks in structural groups, bi-antennary bi-galactosylation (G2) was the strongest biomarker decreased in abundance in CRC cases followed by mono-sialyation and core fucosylation. A decrease in mono-sialylated (S1), α1–6 linked core fucosylated and bi-galactosylated (G2) glycans were all previously observed in lung^10^ and breast^12^ cancer. The increases in G3, G4, S3 and S4 were also consistent with previous lung^10^, periampullary^17^ and breast^12^ cancer *N*-glycan analysis.

Finally, CRP measurements (known to indicate the presence of inflammation) were positively correlated with tri and tetra sialylation and galactosylation (AGP glycans) and negatively correlated with FA2G2 and FA2G2S[6]1 glycans (IgG glycans). This concurs with a previous study, where similar changes were found to occur after major surgery^37^. These glycan changes were also reported previously to be correlated with inflammation^38^, Ulcerative Colitis and Crohn’s Disease^39^.

The tetra-antennary structure A4G4S[3,3,3,3]4 was significantly increased in individuals with high/medium family history (*P* = 1.10E-03 Supplementary Table 6). This glycan is highly branched and requires activity of the GNT-V protein, which is a product of the *MGAT5* gene. This gene has been implicated in ulcerative colitis^40^, gastric^41^ and small cell lung cancer^42^. To the best of our knowledge, this is the first report of a specific glycan level being associated with colorectal cancer family history. Thus, increased levels of A4G4S[3,3,3,3]4 is a marker for not only CRC but high/medium cancer family history too. The ability to identify at-risk individuals before the onset of colorectal cancer through statistical analysis adds further credibility to this investigation, individuals who were sampled at a healthy stage from Finland who later developed CRC also had significantly increased levels of A4G4S[3,3,3,3]4 perhaps indicating that it is a marker for ‘high potential to develop CRC’. Moreover, the at-risk group could be identified with discrimination significantly above random (FINRISK dataset; AUC 0.651). This may also indicate glycans can change in response to heritable mutations in certain genes and requires further investigation.

The integrated plasma UPLC chromatograms contained a much more detailed and complex set of glycan types than our previous IgG study where di-sialylated antennary structures were the largest glycan structure observed^19^. As a consequence, the spectrum of glycan types in this plasma UPLC analysis is greater with tri and tetra sialylated and galactosylated structures assigned. The IgG analysis was based on a similar SOCCS sample set allowing us to compare both studies. Interestingly, when the same glycan was statistically significantly altered in CRC for the both IgG and plasma analysis, only one increase/decrease conflict occurred (Bisecting GlcNAc A2BG2S2 Table 3). This is possible as in IgG analysis the glycoprotein was initially isolated from the plasma samples prior to glycan analysis whereas here the source of glycan abundance comes from multiple glycoproteins. In both sources there was a consistent increase of agalactosylated FA2 and decreases for all structures with two terminal galactoses (A2G2 (GP8), FA2G2 (GP11), FA2BG2 (GP12) and the mono sialic acid structure FA2G2S[6]1. All these glycans are predominately derived from IgG in plasma as summarized in^21^. The only difference between IgG and plasma was the opposite trends for core fucose derived traits (e.g. neutral core fucose peaks increased significantly in IgG but decreased significantly here, see coreFneutral in Table 2). This is not unexpected, since fucose on IgG has a very specific function and is presumably regulated in a different way in B cells and in liver^43^. In addition, IgG is over 90% core-fucosylated whereas in whole plasma, in addition to IgG, there are three other glycoproteins which dominate the glycan profile^22^ with the most abundant peak an afucosylated structure, namely A2G2S[3,6]2.

Discriminating healthy individuals from CRC individuals using simple classification algorithms and relative glycan abundance showed good performance. A logistic regression discrimination model was created in our previous work on IgG^19^ and a simple machine learning model was optimized for this work. Both had very similar model performances ~0.77 AUC suggesting a possible upper limit. Using glycan abundance information (peak area) improved discrimination substantially over the model using only clinical variables (AUC 0.777 vs. 0.613; Fig. 4) suggesting the glycan abundance patterns are much stronger at classifying CRC from healthy control (the same was concluded in the IgG study^19^). Given the good discriminative power of these models, glycan data in combination with other biomarkers/risk factors merit further evaluation in future studies, for example in assessing models to achieve population stratification of risk to guide CRC screening. Perhaps, by using a combination of genomic information, to complement the glycomics evidence, such as single nucleotide polymorphism mutations we could increase the sensitivity and specificity of the model. For instance, a recently highly publicised blood test, CancerSEEK, measured levels of circulating proteins and mutations in cell-free DNA^44^. CancerSEEK could be combined with our orthogonal glycomics method to achieve even greater sensitivity and specificity.

By grouping the patients into stage 1, 2, 3 and 4 of the disease a decrease of F(6)A2G(4)2 and F(6)A2G(4)2S(6)1 across all stages of CRC was observed. However, stage 1 had a unique biomarker signature compared with later stages of the disease. In particular there was no significant increase in the tail region (peaks 36–42) of Stage 1 CRC. Considering this region was correlated with CRP (inflammation) it may be possible that because stage 1 CRC has not yet metastasized inflammation may not be present yet. If this is true the tail region of (peaks 36–42) the chromatogram could be used to highlight a change from stage 1 to more serious, inflamed, stages (2, 3 and 4) but this needs further investigation. This would likely be due to increases in acute phase proteins as the cancer progresses to more serious stages and with them increases in tri and tetra sialylation/galactosylation (i.e. acute phase protein predominately contain these glycans^21^).

This is the first study of this magnitude to examine the complexities of the plasma *N*-glycome and correlate changes to CRC risk. The demographic included the majority of hospitals in Scotland and is therefore broadly representative of the Scottish CRC population. A newly developed high throughput automated robotic platform was utilized to isolate the glycans prior to UPLC-florescence. Overall, the significant changes observed in plasma correlated with changes shown in our recent IgG study adding further merit to this reproducible glycomic technology. Spurious differences between cases and controls were minimized by carrying out the glycan analysis blind to CRC status, plasma was stored at −80 °C until required, and all samples were treated in exactly the same manner.

A limitation of this study was that a single measurement of the glycosylation status from each patient was taken at a given time point. Although unlikely it might be possible that part of the differences we observe may be due to slight technical variation. Environmental factors such as age and obesity are known to influence protein glycosylation. We have taken this into account in our analyses and adjusted accordingly for these contributing factors, however, it remains possible that glycosylation changes observed are due to the occurrence of cancer (reverse causality). We endeavoured to investigate this further by analysing a prospective cohort and we observed that CRC cases could be identified with discrimination significantly above random. In addition, GP40 in the SOCCS study and GP41 in the prospective study both contained increased levels of the glycoform found to be associated with family history. However, the pattern for the remaining glycoforms was different between the two datasets. These differences could be explained by the fact that *N*-glycoforms were measured in the serum in the FINRISK cohort and in the plasma in SOCCS. However, the observed differences in SOCCS can also be a result of the tumour presence (rather than influencing the risk of developing CRC – reverse causality). Finally, the observed differences between CRC cases and controls in SOCCS might be due to the operation for tumour removal that CRC cases had undergone. A recent study examining alterations in the plasma *N*-glycome after cardiac surgery revealed a significant decrease in all sialylated glycans, excluding bi-antennary, di-sialylated structures and tetra-antennary fucosylated structures with the notable exception of SLe^X^ which decreased initially but subsequently increased as the inflammatory response was stimulated^45^. Therefore, comparing our findings with those of the cardiac surgery patients, the decrease in mono-sialyated glycans that we observed in our CRC patients might be due to surgery. However, all other four major glycan alterations observed (including changes in agalactosylation, galactosylation, GlcNAc antennae and core fucosylation) were not observed in cardiac surgery patients and may be specific to CRC disease.

In conclusion, this study has demonstrated significant differences in whole plasma glycome composition between colorectal cancer patients and controls. Although we were not able to detect the majority of these differences in historical samples (possibly due to reverse causality, inadequate study power or different type of sample) the observation that at-risk groups could be segregated from the population via statistical analysis in both sets of cohorts shows potential. *N-*glycome biomarkers in conjunction with other biomarkers/risk factors warrant further investigation in future studies, in assessing models to achieve patient stratification of associated risk and to facilitate CRC screening.

## Methods

### Studies

*The Study of Colorectal Cancer in Scotland (SOCCS)* (1999–2006) is a case control study designed to identify genetic and environmental factors associated with non-hereditary colorectal cancer risk and survival outcomes. Approval for the study was obtained from the MultiCentre Research Ethics Committee for Scotland and Local Research Ethics committee, and all participants gave written informed consent. All methods were performed in accordance with the relevant guidelines and regulations. The study has been described in detail elsewhere^46^. The present study includes a subset of 633 patients with pathologically confirmed colorectal adenocarcinoma and 478 age and gender matched controls. We randomly split the data into a training set used to find statistically significant biomarkers (625 patients, 468 controls) and a validation set used to discriminate CRC from control (8 patients, 10 control). CRC stage was also available for the training set (stage 1 = 110, stage 2 = 199, stage 3 = 231 and stage 4 = 85) and validation set (stage 1 = 2, stage 2 = 2, stage 3 = 2, stage 4 = 2).

Blood was collected post-surgery and transferred to the research centre within 72 hours of sampling. Plasma was prepared by gentle centrifugation of sodium EDTA tubes and 1.5 ml of each participant’s plasma was stored at −80 °C. Each sample was barcoded to ensure analysis was carried out blind. Participants completed a detailed lifestyle questionnaire, a semi-quantitative food frequency (http://www.foodfrequency.org) and a dietary supplements questionnaire. These questionnaires provided information on age, sex, BMI, cancer family history (low, medium or high), smoking (non-smokers, current), physical activity (very low, low, medium or high), non-steroidal anti-inflammatory drugs (NSAIDs) intake (yes, no) and total calorie intake. Plasma levels of C-reactive protein (CRP) were also measured. Plasma glycome composition was analysed in samples collected after CRC diagnosis (for CRC cases) or recruitment (for population controls). To investigate glycan abundance changes against clinical variables such as CRP and family history, a larger unmatched sample set was also used from the SOCCs study, containing 1435 patients and 553 controls.

#### FINRISK

A second serum sample set to investigate risk factors in the Finland population^47^ was used. Population data were searched for patients with incident CRC during 10 years follow-up. Individuals were sampled before developing CRC. 40 individuals (mean age 60 (interquartile range 52.8–68.71)), who developed CRC and no other chronic disease, were identified and included in the study. 80 age and sex matched controls (median age 60.32 (interquartile 54.7–68.8)), who remained healthy during follow-up period, were selected from the same population and serum *N*-glycans were analysed.

### Experimental protocol

A detailed structural analysis and quantification of oligosaccharides in human plasma was performed using a HTP platform for *N*-glycan analysis - UPLC hydrophillic interaction liquid chromatography (HILIC) with fluorescence detection - a technique that separates structures on the basis of their hydrophilicity^23,24^. This well-established protocol was further developed, optimised and automated on a robotic platform^48^. Several bioinformatics tools were developed to assist the analyses, including modelling algorithms, statistical methods and database matching in GlycoBase^49^, a library containing H/UPLC HILIC data for many 2AB labelled *N*-linked and *O*-linked glycans (https://glycobase.nibrt.ie/).

Briefly, the glycoproteins in the plasma samples were denatured and the attached glycans released by PNGase F. A further hydrazide-mediated clean-up step was carried out subsequent to 2AB labelling as described elsewhere^50^. Finally, glycans were immobilized on solid supports and contaminants removed. Alternatively, for the FINRISK replication cohort serum glycans were 2AB labelled immediately after protein deglycosylation and cleaned-up using hydrophilic GHP filter with cold 96% acetonitrile before the UPLC analysis, as previously described^51^. Separation of 2-AB-derivatized *N*-glycans was carried out by UPLC with fluorescence detection on a Waters Acquity UPLC H-Class instrument consisting of a binary or quaternary solvent manager, sample manager, and fluorescence detector under the control of Empower 3 chromatography workstation software (Waters, Milford, MA, USA). A fifth-order polynomial distribution curve was fitted to the dextran ladder to assign glucose unit (GU) values from retention times (using Empower software) as described previously^25^. The GU values obtained for each glycan are reproducible and predictive, and therefore initial structural assignments were possible using recorded GU values in GlycoBase^49^. A detailed protocol is given in the supplementary material including reagents used with their concentrations and incubation times. In addition, the slightly different UPLC conditions for the FINRISK sample set are summarized in the supplementary material.

### Nomenclature for glycan molecules

The nomenclatures used to describe the structures are Oxford nomenclature, with shorthand descriptors as previously described^26^. The *N-*linked glycan core structure is composed of a trimannosylchitobiose core and thus is generally abbreviated M3. Antennae on *N*-glycans are abbreviated A1, A2, A3 or A4 composed of an M3 core with one, two, three and four arm GlcNAc extensions. Further extensions include galactose additions to each antennae (e.g. bi antennae and galactose - A2G2); addition of sialic acids to galactose (e.g. A2G2S2). If fucose is linked to the reducing end GlcNAc of the chitobiose core, generally an F is used prior to the shorthand descriptor to indicate the presence of the core fucose (e.g. FM3, indicating a core fucose attached to the GlcNAc of the chitobiose core of the core *N*-linked structure). Another substitution that can occur is the addition of a GlcNAc to the middle mannose of the chitobiose core, resulting in a bisecting GlcNAc. A structure with this modification is referred to as a bisected glycan, for example a bisected A2G2 can be represented by A2BG2. A comprehensive list of examples is provided in Fig. 1.

### Computational procedures

#### Statistical analysis

Clinical characteristics among patients and controls were compared using a Tukey honest significant difference (HSD) test with ANOVA for all continuous variables. Peak areas are compositional data (presented as a % of the total area under the graph) and therefore the constant-sum constraint (CSC) occurs. The CSC means individual variables do not vary independently, violating common assumptions upon which standard statistical analyses are performed. This was avoided by performing a log transform, *log(Peak*_*i*_*)/(100-Peak*_*i*_*)*, on all peak areas^52^. To calculate the *P*-values we controlled for covariates by creating a linear regression model with available covariates: age, family history and gender for the SOCCS set and age for the FINRISK set. For categorical variables Fisher exact tests were performed. Correction for multiple testing was performed using a simple Bonferroni correction with ANCOVA and Fisher test results considered statistically significant when *P*-values were < 0.05/(number of tests).

#### Peak and clinical feature associations

Peak relative abundances were correlated with continuous variables using Pearson correlation (R) and significance calculated using the t-test (null hypothesis that there is no correlation). Categorical clinical features (e.g. family history) were associated with peaks using Tukey HSD and ANOVA using linear models with age and gender covariates.

#### Classification model

Simple perceptron^53^, models with sigmoidal activation functions were optimized on the peak areas. In order to test the performance of the models the training set was split and a 10-fold cross validation (90% of data for finding model parameters and 10% of data to test the model repeated 10 times) performed. To confirm the accuracy of the model, a perceptron model was optimized on the training set and the optimized model was used to classify CRC on the validation set. Predicting the presence or absence of CRC is a binary classification problem with discriminative power indicated by area under the Receiver Operating Characteristic curve (referred to as AUC)^54^. Prediction models were deemed statistically different by bootstrapping the AUC using the pROC package^55^.

## Electronic supplementary material

Supplementary Information
