# Supplementary material for: Plasma N-glycans in colorectal cancer risk
Source: Sci Rep. 2018 Jun 5;8:8655. doi: 10.1038/s41598-018-26805-7 (PMC5988698; doi:10.1038/s41598-018-26805-7)
Supplement: Supplementary file 1 — Supplementary Information [file 41598_2018_26805_MOESM1_ESM.docx]

**SUPPLEMENTARY FILE FOR**

**Plasma *N*-glycans in colorectal cancer risk**

Margaret Doherty^1,2,~*^, Evropi Theodoratou^3,4,~^, Ian Walsh^5,~^, Barbara Adamczyk^1,6^, Henning Stöckmann^1^, Felix Agakov^7^, Maria Timofeeva^4^, Irena Trbojević-Akmačić^8^, FranoVučković^8^, Fergal Duffy^1^, Ciara A. McManus^1^, Susan M Farrington^4^, Malcolm G Dunlop^4^, Markus Perola^9^, GordanLauc^8, 10#^, Harry Campbell^3,4#^,Pauline M. Rudd^1#^

**1** National Institute for Bioprocessing Research & Training, Dublin, Ireland

**2** Institute of Technology Sligo, Department of Life Sciences, Sligo, Ireland

**3** Centre for Global Health Research, Usher Institute for Population Health Sciences and Informatics, University of Edinburgh, Edinburgh, UK

**4** Colon Cancer Genetics Group, Institute of Genetics and Molecular Medicine, University of Edinburgh and Medical Research Council Human Genetics Unit, Edinburgh, UK

**5** Bioprocessing Technology Institute, Agency for Science, Technology and Research (A*STAR), 20 Biopolis Way, #06-01 Centros, Singapore, 138668, Singapore.

**6** Department of Medical Biochemistry and Cell Biology, Institute of Biomedicine, Sahlgrenska Academy, University of Gothenburg, Gothenburg, Sweden

**7** Pharmatics Limited, Edinburgh Bioquarter, 9 Little France Road, Edinburgh, UK

**8** Genos Glycoscience Research Laboratory, Zagreb, Croatia

**9** Department of Health, The National Institute for Health and Welfare, Helsinki, Finland

**10** University of Zagreb Faculty of Pharmacy and Biochemistry, Zagreb, Croatia

*To whom correspondence should be addressed to: Email: doherty.margaret@itsligo.ie Tel: +353 71930 5726

~These authors contributed equally and are presented in alphabetical order.

# G. Lauc, H. Campbell and P. Rudd contributed equally to this article

**Grant support:** Programme Grant No. C348/A12076 from Cancer Research UK, ET has a Chancellor’s Fellowship from the University of Edinburgh. Glycan analysis was partly supported by European Commission GlycoBioM (contract #259869), IBD-BIOM (contract #305479), HighGlycan (contract #278535), MIMOmics (contract #305280), HTP-GlycoMet (contract #324400), IntegraLife (contract #315997), GastricGlycoExplorer (contract #316929) and GlyCoCan (contract #676412) grants.

**Disclosure of Potential Conflicts of Interest**

Professor Gordan Lauc is founder and owner of Genos Ltd – a private research organization that specialises in high-throughput glycomic analysis. ITA and FV are employees of Genos Ltd. All other authors declare no potential conflicts of interest.

**Author contributions**

Study design – MD, HC, MGD, GL, ET, PR, SMF

Sample provision – HC, MGD, ET, SMF, MP

Scientific analyses – MD, IW, ET, FV, GL, BA, HS, ITA, FD, CM

Statistical analysis – IW, FA, MT

Writing of the manuscript – MD, IW, ET, HC, GL, PR, FA, MT, MGD

# Experimental protocol

**Glycoprotein Denaturation and Glycan Release**

The SOCCS and FINRISK sample set glycan analysis were conducted in different laboratories and hence have slightly different methods which are detailed below.

*Plasma SOCCS sample set:* samples (5 µL) and denaturation buffer (50 μL per well, 100 mM ammonium bicarbonate, 50 mM dithiothreitol, 0.1% sodium dodecyl sulfate) were dispensed into a 96-well V-bottom plate, which was placed on a robotic heater shaker and fully covered and insulated with an anti-evaporation lid. This assembly was incubated at 65°C with agitation at 700 rpm for 20 min. After cooling to room temperature, an iodoacetamide solution (100mM, 10μL per well) was added, and the plate was covered with an anti-evaporation lid and incubated at room temperature with agitation at 700 rpm for 30 min. PNGase F (Prozyme Glyco N-Glycanase, code GKE-5006D, 10μL per well, 0.5 mU in 1 M ammonium bicarbonate, pH 8.0) was added, and the ultrafiltration plate was insulated with an anti-evaporation lid and incubated at 40°C with agitation at 700 rpm for 2 h.

*Serum FinRISK sample set:* Serum samples (10 μL) were denatured with the addition of 20 μL 2% (w/v) SDS. After incubation at 65 °C for 10 min and cooling down to room temperature for 30 min, 10 μL of 4% (v/v) Igepal-CA630 was added. Mixture was shaken for 15 min on a plate shaker. *N*-glycans were released with the addition of 1.2 mU of PNGase F (Promega, Madison, WI, USA) in 10 μL 5× PBS and incubation at 37 °C for 18 hours. Released *N*-glycans were immediately labeled with 2-aminobenzamide (2-AB). The labeling mixture was freshly prepared by dissolving 2-AB (final concentration 19.2 mg/mL) and 2-picoline borane (2-PB, final concentration 44.8 mg/mL) in DMSO and glacial acetic acid mixture (70:30, v/v). To each *N*-glycan sample in the 96-well plate 25 μL of labeling mixture was added and the plate was sealed using adhesive seal. Mixing was achieved by shaking for 10 min, followed by 2 hour incubation at 65 °C.

**Hydrazide-Mediated Glycan Cleanup**

*Plasma SOCCS sample set:* Each well of a 96-well chemically inert filter plate (Millipore Solvinert, hydrophobic polytetrafluoroethylene membrane, 0.45 μm pore size) was washed with 100 μL of methanol (MeOH). UltraLink hydrazide resin (50 μL of a suspension in water, Thermo Scientific) was dispensed to each well. The resin was sequentially washed with MeOH, H_2_O, and acetonitrile (MeCN), and the plate was placed on a heater (70 °C, 10min) to seal the membranes. One hundred eighty microliters of MeCN/acetic acid (98:2) was added to the resin, followed by 20 μL of the glycan solution. The filter plate was incubated with shaking at 700 rpm at 70 °C for 45 min. 50 μLof MeCN/acetic acid (98:2) was added, and shaking was continued at the same temperature for 10 min to disrupt resin aggregates. The resin was washed sequentially with MeOH, guanidine, H2O, triethylamine/MeOH (1:99), and MeOH (200μL per well). Fresh MeOH (180 μL) and acetic anhydride (20 μL) were added, and the plate was incubated for10 min with agitation at 700 rpm. Excess reagent was removed by filtration, and the resins were washed sequentially with MeOH, H_2_O, and MeCN. Acetic acid/MeCN (2:98, 180 μL) and H_2_O (20 μL) were sequentially added, and the plate was incubated at 70 °C with agitation at 700 rpm for 60 min. Fluorescent labeling mix (50 μL, 350 mM 2-aminobenzamide, 1 M sodium cyanoborohydride in acetic acid/dimethylsulfoxide (30:70)) was dispensed into each well, and the plate was incubated at 70 °C with agitation at 700 rpm for 120 min.

**Glycan Solid-Phase Extraction**

*Plasma SOCCS sample set:* The labeling reaction was quenched by the addition of 200 μL of MeCN/H_2_O (95:5). The suspension was transferred to a 2 mL collection plate containing 800 μL of MeCN/H_2_O (95:5) per well, the beads were left to settle, and 200 μL of the supernatant was aspirated and dispensed back into the filter plate. After extensive mixing, the suspension was transferred back into the collection plate. This cycle was repeated once more to ensure a quantitative transfer of the resins. HyperSep Diol SPE cartridges (Thermo Scientific) were washed with 1 mL of MeCN/H_2_O (95:5), 1 mL of H_2_O, and 1mL of MeCN/H_2_O. Next, the beads were suspended and transferred onto the SPE cartridges. A10 min incubation typically led to complete drainage of the solvent by gravity. The SPE cartridges were washed three times with 700 mL of MeCN/H_2_O (95:5). A collection plate was placed inside the robotic vacuum manifold, and the SPE cartridges were washed twice with 200 μL of H_2_O/MeCN (80:20), with an intermittent incubation period of 10 min. The samples were concentrated to dryness and were dissolved in 30 μL of MeCN/H_2_O (70:30) and filtered (Pall Acroprep GHP membrane, 0.45μm pore size). A 10 μL aliquot of the filtrate was analyzed by UPLC.

*Serum FinRISK sample set:* Free label and reducing agent were removed from the samples using hydrophilic interaction liquid chromatography solid phase extraction (HILIC-SPE). Hydrophilic 0.2 μm GHP filter plate (Pall Corporation, Ann Arbor, MI, USA) was used as a stationary phase and solvent in each step was removed using a vacuum manifold (Millipore Corporation, Billerica, MA, USA). All wells were prewashed with 200 μL 70% ethanol (v/v), 200 μL ultra-pure water and 200 μL cold (4 °C) 96% MeCN (v/v). After cooling down to room temperature for 30 min, 700 μL of cold (4 °C) MeCN was added to each sample. The samples were loaded to the wells, and after short incubation subsequently washed 5× 200 μL of (4 °C) 96 % MeCN. Glycans were eluted with 2× 90 μL of ultra-pure water after 15 min shaking at room temperature and combined eluates were stored at -20 °C until usage.

**Ultra Performance Liquid Chromatography (UPLC)**

*Plasma SOCCS sample set:* Separation of 2-AB-derivatized *N*-glycans was carried out by UPLC with fluorescence detection on a Waters Acquity UPLC H-Class instrument consisting of a binary solvent manager, sample manager, and fluorescence detector under the control of Empower 3 chromatography workstation software (Waters, Milford, MA, USA). The HILIC separations were performed using a Waters Ethylene Bridged Hybrid (BEH) Glycan column (150×2.1 mm i.d., 1.7μm BEH particles) with 50 mM ammonium formate (pH 4.4) as solvent A and MeCN as solvent B. The separation was performed using a linear gradient of 70−53% MeCN at 0.56 mL/min in 23 min for serum separation. An injection volume of 10 μL sample prepared in 70% v/v MeCN was used throughout. Samples were maintained at 5°C prior to injection, and the separation temperature was 40 °C. The fluorescence detection excitation emission wavelengths were λ ex= 330 nm and λ em= 420 nm, respectively. The system was calibrated using an external standard of hydrolyzed and 2-AB-labeled glucose oligomers to create a dextran ladder, as described previously ^1^. A fifth-order polynomial distribution curve was fitted to the dextran ladder to assign glucose unit (GU) values from retention times (using Empower software). The chromatograms were integrated into 42 peaks (Peaks 1-42) and the amount of glycans in each peak was expressed as % of total integrated area.

*Serum FINRISK sample set:* As above but Waters Acquity ultra-performance liquid chromatography (UPLC) instrument (Milford, MA, USA) consisting of a quaternary solvent manager, sample manager and a FLR fluorescence detector set with excitation and emission wavelengths of 250 and 428 nm, respectively. The instrument was under the control of Empower 2 software, build 2145 (Waters, Milford, MA, USA). Labelled *N*-glycans were separated on a Waters bridged ethylene hybrid (BEH) Glycan chromatography column, 150 × 2.1 mm, i.d., 1.7 μm BEH particles, with 100 mM ammonium formate, pH 4.4, as solvent A and acetonitrile as solvent B. The separation method used a linear gradient of 70–53% acetonitrile (v/v) at flow rate of 0.561 ml/min in a 25 min analytical run. An injection volume of 20 μL sample prepared in 75% v/v MeCN was used throughout. Samples were maintained at 5 °C before injection, and the separation temperature was 25 °C. The system was calibrated using an external standard of hydrolyzed and 2-AB labelled glucose oligomers from which the retention times for the individual glycans were converted to glucose units. Data processing was performed using an automatic processing method with a traditional integration algorithm after which each chromatogram was manually corrected to maintain the same intervals of integration for all the samples. The chromatograms were integrated into 39 peaks (GP1-GP39) and the amount of glycans in each peak was expressed as % of total integrated area.

**Supplementary Table 1.** Each glycan, its peak membership and GU values. Predominant glycans have a coloured background. **Structure abbreviations: all N-glycans have core sugar sequence consisting of two N-acetylglucosamines (GlcNAc) and three mannose residues; F indicates a core fucose α1–6 linked to the inner GlcNAc; Mx, number (×) of mannose on core GlcNAcs; Ax, number of antenna (GlcNAc) on trimannosyl core; A2, biantennary glycan with both GlcNAcs as β1–2 linked; B, bisecting GlcNAc linked β1–4 to β1–3 mannose; Gx, number of β1–4 linked galactose (G) on antenna; [3]G1 and [6]G1 indicates that the galactose is on the antenna of the α1–3 or α1–6 mannose; Sx, number (×) of sialic acids linked to galactose.**

**Supplementary Table 2.** Individual peak and glycan abundance changes in CRC patients vs. healthy controls. Underlined ***p-*values** show significant changes in CRC compared to control with Bonferroni correction for multiple testing (*p*-values must be < 0.05/42). The median and interquartile ranges (IQR) are shown for each variable. * the difference between the mean peak areas (CRC – control). Iso indicates isomer. GP^NPS^ no predominant structure in which case all are listed. # Tukey test with age, gender and family history adjustments.

| **Peak** | **Predominant**  **glycans** | **CRC (n=625)**  **(median[IQR])** | **Control (n=468)**  **(median[IQR])** | **Δ peak**  **area*** | **P-values^#^** |
| --- | --- | --- | --- | --- | --- |
| GP1 | **FA2** | 2.23 (1.59-3.02) | 2.01 (1.44-2.78) | 0.29 | 1.41E-04 |
| GP2 | FA2B, A2[6]G1, M5 | 1.03 (0.8-1.24) | 1.02 (0.80-1.22) | 0.01 | 7.45E-01 |
| GP3 | A2[6]BG1 | 0.08 (0.06-0.1) | 0.07 (0.06-0.09) | 0.00 | 6.66E-02 |
| GP4 | **FA2[6]G1** | 2.00 (1.56-2.42) | 2.14 (1.70-2.60) | -0.13 | 1.86E-04 |
| GP5 | **FA2[3]G1** | 1.05 (0.82-1.32) | 1.12 (0.90-1.37) | -0.07 | 6.12E-04 |
| GP6 | FA2[6]BG1 | 0.69 (0.55-0.84) | 0.72 (0.60-0.86) | -0.03 | 3.71E-03 |
| GP7 | FA2[3]BG1,  M6 D3 | 0.53 (0.45-0.61) | 0.51 (0.44-0.59) | 0.017 | 1.13E-01 |
| GP8 | **A2[3]G1S[3]1, A2G2** | 0.39 (0.33-0.46) | 0.42 (0.37-0.47) | -0.03 | 3.46E-06 |
| GP9 | A2BG2 | 0.10 (0.08-0.13) | 0.11 (0.08-0.13) | 0.00 | 4.30E-01 |
| GP10 | A2[3]G1S[3]1, FA1G1S[3]1 | 0.13 (0.10-0.17) | 0.14 (0.12-0.17) | -0.00 | 5.69E-02 |
| GP11 | **FA2G2** | 2.40 (1.95-2.95) | 2.83 (2.36-3.36) | -0.42 | 2.00E-16 |
| GP12 | **FA2BG2** | 0.50 (0.42-0.61) | 0.53 (0.46-0.62) | -0.03 | 4.00E-05 |
| GP13 | A2[3]BG1S[6]1, A2[3]BG1S[3]1, M7 D1 | 1.22 (1.09-1.38) | 1.23 (1.12-1.35) | 0.01 | 9.17E-01 |
| GP14 | FA2[3]G1S[3]1, FA2[3]G1S[6]1 | 1.17 (0.98-1.38) | 1.19 (1.04-1.39) | 0.01 | 6.32E-02 |
| GP15 | A2G2S[6]1 | 8.29 (7.58-9.01) | 8.43 (7.80-8.99) | -0.13 | 1.85E-02 |
| GP16 | A2BG2S[6]1 | 0.45 (0.39-0.52) | 0.45 (0.38-0.51) | 0.03 | 1.67E-01 |
| GP17 | A2G2S[3]1 | 0.99 (0.81-1.19) | 1.02 (0.86-1.18) | 0.00 | 1.21E-01 |
| GP18 | **FA2G2S[6]1** | 5.89 (5.08-6.68) | 6.42 (5.57-7.24) | -0.53 | 2.63E-12 |
| GP19 | FA2BG2S[3]1, FA2BG2S[6]1 | 3.20 (2.62-3.82) | 3.19 (2.67-3.81) | 0.00 | 4.19E-01 |
| GP20^NPS^ | **A2F1G2S[3]1, A2F1G2S[6]1** | 1.16 (0.99-1.33) | 1.27 (1.12-1.42) | -0.09 | 3.89E-06 |
| GP21 | A2G2S[3,6]2^iso^ | 3.42 (2.93-3.91) | 3.31 (2.9-3.77) | 0.10 | 2.11E-01 |
| GP22 | M9 | 0.84 (0.72-0.96) | 0.85 (0.74-0.97) | 0.18 | 2.00E-01 |
| GP23 | A2G2S[3,6]2^iso^ | 29.35 (27.38-31.45) | 29.79 (27.81-31.89) | 0.46 | 1.85E-02 |
| GP24^NPS^ | **FA3G3S[3]1, FA3G3S[6]1,FA3BG3S[3]1,A2BG2S[3,3]2, A2BG2S[3,6]2,A2BG2S[6,6]2** | 0.94 (0.82-1.06) | 0.9 (0.80-1.00) | 0.07 | 6.05E-04 |
| GP25 | FA2G2S[3,6]2 | 5.73 (5.02-6.57) | 6.005 (5.09-6.79) | -0.19 | 4.04E-02 |
| GP26 | FA2BG2S[3,6]2, FA2BG2S[6,6]2 | 3.3 (2.69-3.95) | 3.34 (2.85-4) | -0.09 | 3.68E-02 |
| GP27 | **A3G3S[3,6]2, A3BG3S[3,6]2** | 1.5 (1.27-1.80) | 1.38 (1.18-1.61) | 0.14 | 1.74E-07 |
| GP28 | A4G4S[3]1 | 0.45 (0.40-0.50) | 0.44 (0.40-0.49) | 0.02 | 7.84E-03 |
| GP29 | A3G3S[3,3]2 | 1.37 (1.17-1.59) | 1.34 (1.17-1.53) | 0.01 | 5.40E-01 |
| GP30 | A3G3S[3,3,3]3 | 0.60 (0.48-0.75) | 0.57 (0.48-0.69) | 0.04 | 2.20E-03 |
| GP31 | A3G3S[3,3,6]3 | 1.06 (0.86-1.29) | 0.99 (0.86-1.17) | 0.06 | 1.85E-02 |
| GP32 | **FA3G3S[3,3,3]3** | 0.20 (0.17-0.24) | 0.19 (0.17-0.22) | 0.07 | 1.76E-05 |
| GP33 | A3G3S[3,3,6]3 | 6.55 (5.50-7.85) | 6.45 (5.37-7.51) | 0.19 | 4.47E-01 |
| GP34 | FA3G3S[3,3,6]3, FA3G3S[3,6,6]3 | 0.6 (0.49-0.78) | 0.59 (0.48-0.73) | 0.04 | 7.95E-03 |
| GP35 | A3F1G3S[3,3,3]3 | 1.87 (1.53-2.23) | 1.83 (1.55-2.15) | 0.04 | 2.85E-01 |
| GP36 | A4G4S[3,3,3]3 | 3.64 (2.61-4.82) | 3.38 (2.53-4.19) | 0.42 | 3.43E-04 |
| GP37 | **A4G4S[3,3,6]3, A4G4S[3,6,6]3** | 0.48 (0.41-0.57) | 0.45 (0.39-0.52) | 0.04 | 1.71E-08 |
| GP38 | **A4F1G3S[3,3,6]3, A4F1G3S[3,6,6]3** | 0.46 (0.33-0.63) | 0.41 (0.32-0.51) | 0.08 | 1.30E-08 |
| GP39 | **A4G4S[3,3,3,3]4^iso^** | 0.53 (0.46-0.64) | 0.48 (0.43-0.55) | 0.07 | 2.00E-16 |
| GP40 | **A4G4S[3,3,3,3]4^iso^** | 0.30 (0.23-0.4) | 0.26 (0.21-0.32) | 0.05 | 3.21E-10 |
| GP41 | **A4G4S[3,3,3,6]4^iso,^ A4G4S[3,3,3,6]4^iso^** | 0.66 (0.49-0.83) | 0.57 (0.46-0.70) | 0.11 | 3.99E-08 |
| GP42 | **A4G4S[3,3,3,3]4^iso^,A4F1G4S[3,3,6,6]4** | 0.46 (0.34-0.62) | 0.4 (0.29-0.53) | 0.12 | 1.19E-01 |

**Supplementary Table 3. FINRISK statistics.** Individual peak and glycan abundance changes in at risk group (individuals who later developed CRC) vs. no risk controls (individuals who did not develop CRC). Individuals were all sampled when healthy. Underlined ***p-values*** show significant changes in CRC compared to control with Bonferroni correction for multiple testing. The median and interquartile ranges (IQR) are shown for each variable. * the difference between the mean peak areas (at risk – control). Iso indicates isomer. GP^NPS^ no predominant structure in which case all are listed. # Tukey test with age adjustments.

| **Peak** | **Predominant**  **glycans** | **At risk (n=40)**  **(median[IQR])** | **Control (n=80)**  **(median[IQR])** | **Δ peak**  **area*** | **P-values^#^** |
| --- | --- | --- | --- | --- | --- |
| GP1 | FA2 | 5.8 ( 4.36 - 7.81 ) | 5.89 ( 4.33 - 7.49 ) | -0.09 | 4.79E-01 |
| GP2 | FA2B, A2[6]G1, M5 | 2.54 ( 2.1 - 2.95 ) | 2.55 ( 2.09 - 2.94 ) | -0.01 | 7.19E-01 |
| GP3 | A2[6]BG1 | 0.11 ( 0.09 - 0.13 ) | 0.11 ( 0.09 - 0.13 ) | 0.00 | 9.46E-01 |
| GP4 | FA2[6]G1 | 4.39 ( 3.7 - 5.1 ) | 4.43 ( 3.79 - 4.96 ) | -0.04 | 6.30E-01 |
| GP5 | FA2[3]G1 | 2.19 ( 1.71 - 2.53 ) | 2.3 ( 1.98 - 2.57 ) | -0.11 | 2.33E-01 |
| GP6 | FA2[6]BG1 | 1.6 ( 1.3 - 1.84 ) | 1.59 ( 1.36 - 1.72 ) | 0.00 | 7.21E-01 |
| GP7 | FA2[3]BG1, M6 D3 | 1.16 ( 1.04 - 1.27 ) | 1.15 ( 1.04 - 1.23 ) | 0.01 | 6.42E-01 |
| GP8 | A2[3]G1S[3]1, A2G2 | 0.9 ( 0.79 - 0.97 ) | 0.93 ( 0.85 - 0.99 ) | -0.03 | 3.35E-01 |
| GP9 | A2BG2 | 0.12 ( 0.1 - 0.13 ) | 0.11 ( 0.1 - 0.13 ) | 0.00 | 9.93E-01 |
| GP10 | A2[3]G1S[3]1, FA1G1S[3]1, FA2G2 | 3.89 ( 3.32 - 4.35 ) | 3.97 ( 3.07 - 4.53 ) | -0.08 | 7.95E-01 |
| GP11 | FA2BG2 | 0.89 ( 0.74 - 1.01 ) | 0.9 ( 0.77 - 1.02 ) | 0 | 8.74E-01 |
| GP12 | A2[3]BG1S[6]1, A2[3]BG1S[3]1, M7 D1 | 1.04 ( 0.95 - 1.11 ) | 1.06 ( 0.96 - 1.16 ) | -0.02 | 4.32E-01 |
| GP13 | FA2[3]G1S[3]1, FA2[3]G1S[6]1 | 0.85 ( 0.73 - 1.02 ) | 0.88 ( 0.78 - 0.97 ) | -0.03 | 3.49E-01 |
| GP14 | A2G2S[6]1 | 9.12 ( 8.46 - 9.62 ) | 9.5 ( 9.01 - 9.89 ) | -0.38 | 1.78E-02 |
| GP15 | A2BG2S[6]1 | 0.43 ( 0.36 - 0.46 ) | 0.42 ( 0.36 - 0.46 ) | 0.01 | 7.29E-01 |
| GP16 | A2G2S[3]1, FA2G2S[6]1 | 5.48 ( 5.07 - 5.78 ) | 5.5 ( 4.85 - 6.13 ) | -0.01 | 9.23E-01 |
| GP17 | FA2BG2S[3]1, FA2BG2S[6]1 | 2.46 ( 2.1 - 2.93 ) | 2.4 ( 1.94 - 2.79 ) | 0.06 | 6.13E-01 |
| GP18 | A2G2S[3,6]2^iso^ | 3.41 ( 3.04 - 3.74 ) | 3.41 ( 3 - 3.76 ) | -0.01 | 9.42E-01 |
| GP19 | M9 | 1.08 ( 1 - 1.14 ) | 1.11 ( 1.03 - 1.18 ) | -0.03 | 1.88E-01 |
| GP20 | A2G2S[3,6]2^iso^ | 26.32 (24.79 - 27.15 ) | 25.99 ( 24.53 - 27.37 ) | 0.34 | 5.08E-01 |
| GP21 | FA3G3S[3]1, FA3G3S[6]1,FA3BG3S[3]1,A2BG2S[3,3]2, A2BG2S[3,6]2,A2BG2S[6,6]2 | 0.62 ( 0.57 - 0.68 ) | 0.63 ( 0.58 - 0.67 ) | -0.01 | 3.18E-01 |
| GP22 | FA2G2S[3,6]2 | 4.41 ( 4.00 - 4.83 ) | 4.34 ( 3.72 - 4.88 ) | 0.06 | 6.62E-01 |
| GP23 | FA2BG2S[3,6]2, FA2BG2S[6,6]2 | 2.71 ( 2.18 - 3.19 ) | 2.75 ( 2.13 - 3.21 ) | -0.04 | 7.21E-01 |
| GP24 | A3G3S[3,6]2, A3BG3S[3,6]2 | 1.53 ( 1.30 - 1.72 ) | 1.53 ( 1.30 - 1.72 ) | 0.02 | 5.88E-01 |
| GP25 | A4G4S[3]1 | 0.18 ( 0.16 - 0.19 ) | 0.19 ( 0.17 - 0.21 ) | -0.01 | 5.90E-02 |
| GP26 | A3G3S[3,3]2 | 1.48 ( 1.34 - 1.6 ) | 1.45 ( 1.26 - 1.61 ) | 0.03 | 4.48E-01 |
| GP27 | A3G3S[3,3,3]3 | 0.7 ( 0.52 - 0.85 ) | 0.8 ( 0.67 - 0.9 ) | -0.09 | 5.16E-02 |
| GP28 | A3G3S[3,3,6]3 | 0.72 ( 0.58 - 0.81 ) | 0.68 ( 0.54 - 0.79 ) | 0.04 | 1.30E-01 |
| GP29 | FA3G3S[3,3,3]3 | 0.19 ( 0.17 - 0.21 ) | 0.18 ( 0.15 - 0.21 ) | 0.01 | 7.47E-02 |
| GP30 | A3G3S[3,3,6]3 | 5.34 ( 4.51 - 5.92 ) | 4.88 ( 4.01 - 5.73 ) | 0.47 | 1.91E-02 |
| GP31 | FA3G3S[3,3,6]3, FA3G3S[3,6,6]3 | 0.51 ( 0.4 - 0.58 ) | 0.45 ( 0.36 - 0.52 ) | 0.06 | 2.24E-02 |
| GP32 | A3F1G3S[3,3,3]3 | 1.46 ( 1.26 - 1.68 ) | 1.41 ( 1.17 - 1.61 ) | 0.05 | 2.87E-01 |
| GP33 | A4G4S[3,3,3]3 | 2.74 ( 2.01 - 3.22 ) | 3.03 ( 2.5 - 3.50 ) | -0.29 | 1.41E-01 |
| GP34 | A4G4S[3,3,6]3, A4G4S[3,6,6]3 | 0.39 ( 0.33 - 0.42 ) | 0.36 ( 0.32 - 0.39 ) | 0.02 | 1.30E-01 |
| GP35 | A4F1G3S[3,3,6]3, A4F1G3S[3,6,6]3 | 0.35 ( 0.28 - 0.39 ) | 0.37 ( 0.29 - 0.43 ) | -0.02 | 4.02E-01 |
| GP36 | A4G4S[3,3,3,3]4^iso^ | 0.49 ( 0.42 - 0.57 ) | 0.48 ( 0.43 - 0.53 ) | 0.01 | 4.73E-01 |
| **GP37** | **A4G4S[3,3,3,3]4^iso^** | 0.50 ( 0.41 - 0.57 ) | 0.43 ( 0.35 - 0.51 ) | 0.07 | 1.10E-03 |
| GP38 | A4G4S[3,3,3,6]4^iso,^ A4G4S[3,3,3,6]4^iso^ | 0.99 ( 0.85 - 1.10 ) | 0.90 ( 0.81 - 0.98 ) | 0.09 | 4.83E-03 |
| GP39 | A4G4S[3,3,3,3]4^iso^, A4F1G4S[3,3,6,6]4 | 0.87 ( 0.68 – 1.00 ) | 0.90 ( 0.74 - 1.01 ) | -0.03 | 4.20E-01 |

**Supplementary Table 4. Derived traits by simply summing peak areas with certain characteristics.** * Approximation = predominant glycans assumed to be only contribution to abundance and therefore the formula.

| Derived feature | Description | Formula |
| --- | --- | --- |
| G0 | Total % area containing 0 galactose | GP01 |
| G1 | Total % area containing 1 galactose | GP02+GP03+GP04+GP05+GP06+GP07+GP08+GP10+GP13+GP14 |
| G2 | Total % area containing 2 galactose | GP07+GP11+GP12+GP15+GP16+GP18+GP19+GP20+GP21+GP23+GP25+GP26 |
| G3 | Total % area containing 3 galactose | GP27+GP29+GP30+GP31+GP32+GP33+GP35+GP38 |
| G4 | Total % area containing 4 galactose | GP28+GP36+GP37+GP39+GP40+GP41+GP42 |
| S0neutral | Total % area containing 0 sialic acid | GP01+GP02+GP03+GP04+GP05+GP06+GP07+GP09+GP11+GP12+GP22 |
| S1 | Total % area containing 1 sialic acid | GP08+GP10+GP13+GP14+GP15+GP16+GP17+GP18+GP19+GP20+GP28 |
| S2 | Total % area containing 2 sialic acid | GP21+GP23+GP25+GP26+GP27+GP29 |
| S3 | Total % area containing 3 sialic acid | GP30+GP31+GP32+GP33+GP34+GP36+GP37+GP38 |
| S4 | Total % area containing 4 sialic acid | GP39+GP40+GP41+GP42 |
| CoreFall | Total % area of all core fucose glycans | GP01+GP02+GP04+GP05+GP06+GP07+GP10+GP11+GP12+GP14+GP18+GP25+GP26+GP32+GP34 |
| CoreFneutral | Total % area of core fucose glycans without a sialic acid | GP01+GP02+GP04+GP05+GP06+GP07+GP11+GP12+GP14+GP18 |
| CoreFneutralG1G2 | Total % area of core fucose glycans without sialic acid but must have 1 or 2 galactose | GP04+GP05+GP11+GP12 |
| Ball | Total % area of all bisecting GlcNAc glycans | GP02+GP03+GP04+GP06+GP07+GP09+GP12+GP13+GP16+GP19+GP26+GP27 |
| Bneutral | Total % area of bisecting GlcNAcs glycans without a sialic acid | GP02+GP03+GP04+GP06+GP07+GP09+GP12 |
| OuterF | Total % area of all outer fucose glycans | GP20+GP35+GP38+GP42 |
| Tail | The area of peaks 36-42 inclusive. Also contains the SLex epitope. | GP36+GP37+GP38+GP39+GP40+GP41+GP42 |

**Supplementary Table 5.** Each of the derived traits which were deemed statistically significant in Table 2 main text and a corresponding example glycan also deemed statistically significant in Supplementary Table 2.

| **General biomarker** | **Related specific glycans significantly altered from Supplementary Table 2** | **Decreased/increased** | ***P*-value(s)** |
| --- | --- | --- | --- |
| Agalactosylation (G0) | FA2 | Increased in CRC | 1.41E-04 |
| Tri & tetra galactosylation (G3 & G4) | A3G3S[3,6]2, A3BG3S[3,6]2, FA3G3S[3,3,3]3, A4G4S[3,3,6]3, A4G4S[3,6,6]3, A4F1G3S[3,3,6]3, A4F1G3S[3,6,6]3, A4G4S[3,3,3,3]4^iso^, A4G4S[3,3,3,3]4^iso^, A4G4S[3,3,3,6]4^iso^, A4G4S[3,3,3,3]4^iso^, A4F1G4S[3,3,6,6]4 | Increased in CRC | 6.25E-10, 2.87E-08 |
| Tri & tetra sialyation (S3 & S4) | FA3G3S[3,3,3]3, A4G4S[3,3,6]3, A4G4S[3,6,6]3, A4F1G3S[3,3,6]3, A4F1G3S[3,6,6]3, A4G4S[3,3,3,3]4^iso^, A4G4S[3,3,3,3]4^iso^, A4G4S[3,3,3,6]4^iso^, A4G4S[3,3,3,3]4^iso^, A4F1G4S[3,3,6,6]4 | Increased in CRC | 2.37E-11, 0.00000274 |
| Tail region | FA3G3S[3,3,3]3, A4G4S[3,3,6]3, A4G4S[3,6,6]3, A4F1G3S[3,3,6]3, A4F1G3S[3,6,6]3, A4G4S[3,3,3,3]4^iso^, A4G4S[3,3,3,3]4^iso^, A4G4S[3,3,3,6]4^iso^, A4G4S[3,3,3,3]4^iso^, A4F1G4S[3,3,6,6]4 | Increased in CRC | 9.94E-09 |
| Bi-galactosylation (G2) | FA2G2, FA2BG2, A2G2, FA2G2S[6]1, A2F1G2S[3]1, A2F1G2S[6]1 | Decreased in CRC | 5.00E-08 |
| Mono-sialylation (S1) | FA2G2S[6]1, A2F1G2S[3]1, A2F1G2S[6]1 | Decreased in CRC | 1.80E-04 |
| Neutral core fucose (coreFneutral) | FA2BG2, FA2G2, FA2[3]G1, FA2[6]G1, FA2 | Decreased in CRC | 1.51E-03 |
| Neutral core fucose with one or two galactose (coreFneutralG1G2) | FA2BG2, FA2G2, FA2[3]G1, FA2[6]G1 | Decreased in CRC | 3.57E+00 |

**Supplementary Table 6.** Significant glycan peaks which are altered by descriptive features on the larger set of 1435 patients and 553 controls. Continuous features (BMI, Age and CRP): 95% confidence interval (R 95 CI) on Pearson correlation coefficient (R) and below it the t-test *P*-value (alternative hypothesis: true correlation is not equal to 0). Categorical feature family history p-values from an ANOVA Tukey HSD test with Bonferroni correction. In tests where there was no descriptive variable that individual was removed. NS: not significant. In bold strong associations defined as CI above 0.3 R for continuous and *P*-value < .05 Bonferroni corrected.

| **Feature** | **Predominant**  **glycan structure(s)** | **BMI**  **R**  **[R 95 CI]**  **(*P*-value)** | **CRP**  **R**  **[R 95 CI]**  **(*P*-value)** | **Family history** |
| --- | --- | --- | --- | --- |
| GP1 | FA2 | NS | NS | NS |
| GP4 | FA2[6]G1 | NS | -0.21  [-0.23, -0.14]  (2.2E-16) | NS |
| GP5 | FA2[3]G1 | NS | -0.17  [-0.22, -0.11]  (1.53E-15) | NS |
| GP8 | A2[3]G1S[3]1, A2G2 | -0.13  [-0.18, -0.08]  (6.98E-08) | -0.16  [-0.21, -0.11]  (1.5E-13) | NS |
| GP11 | FA2G2 | -0.10  [-0.15, -0.05]  (1.31E-05) | **-0.27**  **[-0.32, -0.22]**  **(2.2E-16)** | NS |
| GP12 | FA2BG2 | -0.09  [-0.15, -0.04]  (9.44E-05) | -0.21  [-0.27, -0.17]  (2.2E-16) | NS |
| GP18 | FA2G2S[6]1 | NS | **-0.27**  **[-0.32, -0.23]**  **(2.2E-16)** | NS |
| GP20^NPS^ | A2F1G2S[3]1, A2F1G2S[6]1 | -0.12  [-0.17, -0.07]  (6.2E-06) | **-0.24**  **[-0.29,-0.19]**  **(2.2E-16)** |  |
| GP24^NPS^ | FA3G3S[3]1, FA3G3S[6]1,FA3BG3S[3]1,A2BG2S[3,3]2, A2BG2S[3,6]2,A2BG2S[6,6]2 | NS | 0.11  [0.07, 0.17]  (8.07E-05) | NS |
| GP27 | A3G3S[3,6]2, A3BG3S[3,6]2 | -0.15  [-0.20, -0.10]  (1.045E-09) | NS | NS |
| GP32 | FA3G3S[3,3,3]3 | NS | NS | NS |
| GP36 | A4G4S[3,3,3]3 | NS | **0.45**  **[0.40, 0.49]**  **(2.2E-16)** | NS |
| GP37 | A4G4S[3,3,6]3, A4G4S[3,6,6]3 | 0.20  [0.14, 0.24]  (8.7E-14) | **0.36**  **[0.31, 0.41]**  **(2.2E-16)** | NS |
| GP38 | A4F1G3S[3,3,6]3, A4F1G3S[3,6,6]3 | NS | **0.49**  **[0.45, 0.53]**  **(2.2E-16)** | NS |
| GP39 | A4G4S[3,3,3,3]4^iso^ | NS | **044**  **[0.40, 0.48]**  **(2.2E-16)** | NS |
| GP40 | A4G4S[3,3,3,3]4^iso^ | -0.12  [-0.17, -0.07]  (1.69E-07) | **0.23**  **[0.18, 0.29]**  **(2.2E-16)** | **1.65E-03** |
| GP41 | A4G4S[3,3,3,6]4^iso,^ A4G4S[3,3,3,6]4^iso^ | NS | **0.44**  **[0.40, 0.49]**  **2.2E-16** | NS |
| GP42 | A4G4S[3,3,3,3]4^iso^, A4F1G4S[3,3,6,6]4 | NS | **0.56**  **[0.52, 0.61]**  **(2.2E-16)** | NS |

**Supplementary Table 7.** Discrimination performance on each stage using all peak areas in a perceptron model. 10-fold cross validation on the training set (625 CRC vs. 468 controls).

| Stage vs. control | AUC | Sensitivity | Specificity | #Cancer | #Control |
| --- | --- | --- | --- | --- | --- |
| One | 0.7770 | 0.3482 | 0.9477 | 110 | 468 |
| Two | 0.7810 | 0.3681 | 0.9477 | 199 | 468 |
| Three | 0.7400 | 0.2790 | 0.9477 | 231 | 468 |
| Four | 0.8400 | 0.5287 | 0.9477 | 85 | 468 |

**Supplementary Table 8.** Probability of CRC for individuals in the validation set (8 cancer cases vs. 10 healthy control). The probability was produced from a perceptron model optimized on the training set. The cut-off threshold was found at a 95% specificity on the training set. Clinical variables include: Age, Gender, smoking status, NSAIDs, BMI and physical activity. Colors red mark the predicted and actual CRC cases and green mark the predicted and actual healthy control.

|  | |  | | | Threshold | | |  |
| --- | --- | --- | --- | --- | --- | --- | --- | --- |
|  | |  |  |  | **0.89** | **0.90** | **0.69** |  |
| Age | **Gender** | | **Family History** | **Stage** | **Probability**  **Using all peaks only** | **Probability using all peaks + clinical features** | **Probability using CRP only** | **CRC/Control** |
| 47 | M | | Low | 1 | 0.53245932 | 0.070558393 | 0.460330586 | CRC |
| 54 | M | | Med. | 1 | 0.94712192 | 0.94350599 | 0.407348271 | CRC |
| 56 | F | | Low | 2 | 0.38246826 | 0.06636277 | 0.404094387 | CRC |
| 37 | F | | Med. | 2 | 0.70823832 | 0.998345412 | 0.407348271 | CRC |
| 56 | M | | Low | 3 | 0.59095047 | 0.145832732 | 0.436967203 | CRC |
| 56 | M | | Low | 3 | 0.98414005 | 0.999999023 | 0.996108523 | CRC |
| 52 | F | | Low | 4 | 0.95449443 | 0.987512031 | 0.424833337 | CRC |
| 58 | M | | Low | 4 | 0.97191761 | 0.999877106 | 0.996643577 | CRC |
| 54 | M | | Low | - | 0.13360247 | 0.004332045 | 0.410610302 | Control |
| 55 | M | | Low | - | 0.08189839 | 0.033955584 | 0.450290235 | Control |
| 55 | F | | Low | - | 0.02354871 | 0.002769477 | 0.406262725 | Control |
| 47 | M | | Low | - | 0.01822226 | 0.000331934 | 0.404094387 | Control |
| 48 | F | | Low | - | 0.03085042 | 0.035827442 | 0.406262725 | Control |
| 51 | M | | Low | - | 0.04698936 | 0.003488607 | 0.405178093 | Control |
| 56 | F | | Low | - | 0.07768438 | 0.001683494 | 0.420442591 | Control |
| 55 | M | | Low | - | 0.75886291 | 0.532909912 | 0.528816778 | Control |
| 50 | F | | Low | - | 0.82241068 | 0.995358991 | 0.46928271 | Control |
| 45 | F | | Low | - | 0.04740895 | 0.001615516 | 0.406262725 | Control |

**
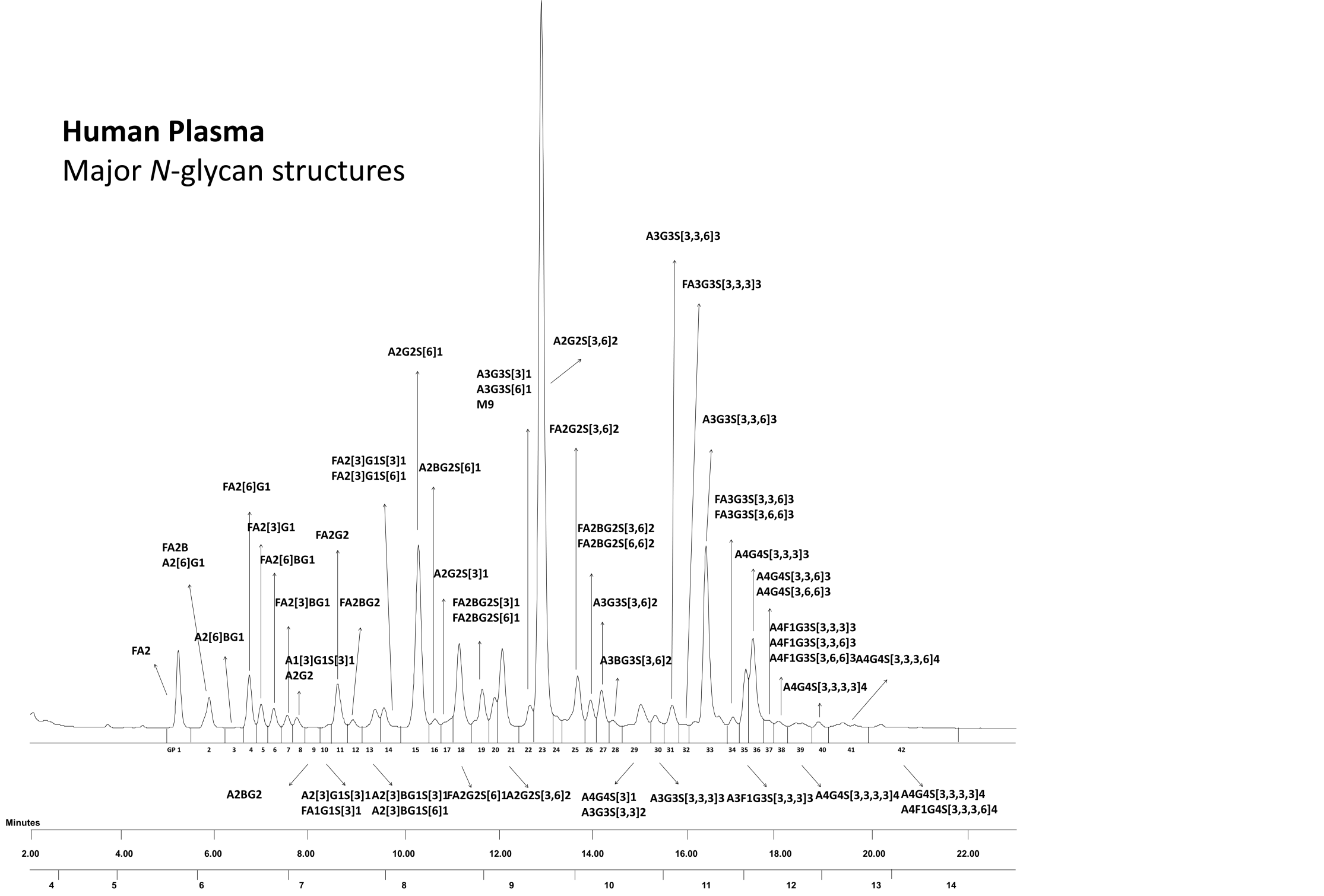
**

**Supplementary Figure 1.** A representative human plasma *N*-glycosylation chromatogram and peak assignments from the CRC cohort. The Oxford nomenclature has been used to annotate individual glycan structures where A represents the number of antennae present, F indicates the fucose, B indicates the presence of a bisecting *N*-acetylglucosamine, G represents galactoses and S denotes sialic acids.

**Supplementary Figure 2.** Significant peak area difference for CRC associated GP40 between low and medium/high family on 1636 low individuals and 253 high/medium individuals with family history assigned.

**REFERENCES**

(1) Royle, L.; Campbell, M. P.; Radcliffe, C. M.; White, D. M.; Harvey, D. J.; Abrahams, J. L.; Kim, Y. G.; Henry, G. W.; Shadick, N. A.; Weinblatt, M. E.; Lee, D. M.; Rudd, P. M.; Dwek, R. A. *Anal Biochem* **2008**, *376* (1), 1–12.

(2) Harvey, D. J.; Merry, A. H.; Royle, L.; Campbell, M. P.; Dwek, R. A.; Rudd, P. M. *Proteomics*. 2009, pp 3796–3801.
